# Nitric Oxide‐Releasing Catheters with Phenol‐Amine Catalytic Coatings for Improved Anti‐Inflammatory Performance

**DOI:** 10.1002/adhm.202500457

**Published:** 2025-08-29

**Authors:** Qingqing Fan, Shu Geng, Tanveer A. Tabish, Kang Lin, Yiyi Yin, Siti Nur Asyura Adzlan, Federico Mazur, Rona Chandrawati

**Affiliations:** ^1^ School of Chemical Engineering and Australian Centre for Nanomedicine (ACN) The University of New South Wales Sydney NSW 2052 Australia; ^2^ Department of Chemical Engineering The University of Melbourne Parkville VIC 3010 Australia; ^3^ Division of Cardiovascular Medicine Radcliffe Department of Medicine British Heart Foundation (BHF) Centre of Research Excellence University of Oxford Headington Oxford OX3 7BN UK

**Keywords:** catheters, nitric oxide, selenocystamine, S‐nitrosoglutathione, tannic acid

## Abstract

Nitric oxide (NO) is a signaling molecule critical for vasodilation, bacterial inhibition, and inflammation modulation, making it valuable for medical devices, such as catheters. However, existing NO‐releasing catheters face challenges, including maintaining stable NO release over time and complex manufacturing processes. In this study, a robust nanocoating made from tannic acid (TA) and selenocystamine (SeCA) is presented, designed to catalytically generate NO from *S*‐nitrosoglutathione in the presence of glutathione. Surface characterization techniques confirmed successful coating formation, with optimal NO generation achieved at a 1:4 TA:SeCA molar ratio. The coated catheters maintained over 96% viability of human coronary artery smooth muscle cells (HCASMCs) after 72 h. It has been demonstrated that the modified catheters significantly increased endogenous NO production in HCASMCs from intracellular *S*‐nitrosothiols, highlighting their potential to support vascular function. The coating sustained NO release at 7 × 10^−10^ mol cm^−2^ min^−1^ for at least 3 days, exceeding the typical release rate of healthy endothelium. Notably, the coating effectively reduced pro‐inflammatory cytokine (TNF‐α and IL‐6) production in RAW 264.7 macrophages upon lipopolysaccharide stimulation, demonstrating anti‐inflammatory effects. This material‐driven approach simplifies NO delivery and offers an effective strategy to enhance catheter efficacy to support vascular function and minimize inflammation.

## Graphical abstract

Tannic acid‐selenocystamine (TA‐SeCA) nanocoating enables stable, sustained catalytic nitric oxide (NO) release on catheter surfaces. This one‐step phenol‐amine coating improves catheter functionality by enhancing endogenous NO production, supporting vascular function, and reducing inflammation.

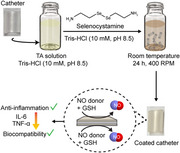

## 1 Introduction

Reliable vascular access is fundamental to modern medical care.^[^ ^1^ ^]^ The growing prevalence of life‐threatening conditions and the increasing need for minimally invasive procedures to diagnose and treat chronic diseases have driven significant expansion in the intravascular catheter market. Valued at USD 10.07 billion in 2024, the market is projected to reach USD 18.12 billion by 2032.^[^ ^2^ ^]^ Despite their essential role, intravascular catheters carry a notable risk of complications, particularly catheter‐related bloodstream infections (CRBSIs).^[^ ^3^ ^]^ CRBSI rates in developed countries typically range from 0.2 to 1.4 per 1000 catheter‐days, while rates tend to be significantly higher in developing countries.^[^ ^4^, ^5^ ^]^ Each new episode of CRBSI increases the risk of septicemia by 4% to 14% and raises the risk of death by 12% to 25%.^[^ ^6^ ^]^ In addition to CRBSI, catheters can also trigger localized inflammation at the insertion site, further complicating patient outcomes.^[^ ^7^ ^]^ These issues not only extend hospital stays but also significantly increase healthcare costs, highlighting the need for advanced therapeutic strategies to improve catheter safety and functionality.

Current strategies to address catheter‐related complications are still limited in effectively preventing infections and inflammation. Although distinct in nature, these complications pose significant risks and require innovative therapeutic approaches. Bloodstream infections often result from bacterial colonization of catheters,^[^ ^8^ ^]^ while local inflammation typically arises from the immune system's reaction to the catheter itself.^[^ ^7^ ^]^ This immune response is associated with increased levels of pro‐inflammatory cytokines, such as tumour necrosis factor alpha (TNF‐α) and interleukin 6 (IL‐6), which play critical roles in mediating infection and inflammation.^[^ ^9^, ^10^ ^]^ To improve therapeutic outcomes and mitigate both infection and inflammation, catheters are commonly treated with lock solutions that contain high concentrations of antibiotics^[^ ^11^, ^12^ ^]^ and anti‐inflammatory agents.^[^ ^13^ ^]^ Although some clinical trials have reported success,^[^ ^12^ ^]^ other studies have highlighted significant limitations. Antibiotic‐based methods carry the risk of developing drug resistance, while anti‐inflammatory drugs may have limited efficacy in preventing persistent inflammation and can lead to undesirable side effects with prolonged use.^[^ ^14^ ^]^ As such, there is a growing interest in alternative approaches.

One such alternative is nitric oxide (NO), a versatile cell‐signaling molecule produced endogenously through the oxidation of L‐arginine by the heme‐containing enzyme NO synthase (NOS).^[^ ^15^ ^]^ NO plays a crucial role in regulating vasodilation, inhibiting bacterial infections, and modulating inflammation.^[^ ^16^ ^]^ However, its short half‐life and rapid diffusion necessitate the incorporation of NO donors, such as *S*‐nitrosothiols (RSNOs) or diazeniumdiolates (NONOates), into catheters for effective delivery.^[^ ^17^, ^18^, ^19^, ^20^ ^]^ For instance, Wo et al. developed CarboSil catheters containing *S*‐nitroso‐*N*‐acetylpenicillamine (SNAP) that released NO at physiological levels for at least 14 days, reducing *Staphylococcus epidermidis* and *Pseudomonas aeruginosa* biofilms by 2 and 2.5 log units, respectively.^[^ ^18^ ^]^ Another approach to fabricating NO‐releasing catheters employed Elast‐eon E2As polymers combined with diazeniumdiolated dibutylhexanediamine (DBHD/NONO) and poly(lactic‐co‐glycolic acid), achieving controlled NO release over a 9‐day period in a rabbit model, resulting in a sevenfold reduction in thrombosis and a 95% reduction in bacterial adhesion.^[^ ^17^ ^]^ Although SNAP and NONOates enable long‐term NO release, both donors are inherently unstable.^[^ ^16^, ^21^ ^]^ Storage conditions (e.g., pH and temperature) and sterilization methods (e.g., autoclaving or UV‐light exposure) can compromise their stability.^[^ ^22^ ^]^

To overcome these limitations, recent research has focused on developing more stable and efficient NO‐releasing systems. Seleno compounds, such as selenocystamine, have emerged as promising candidates due to their ability to catalyze the decomposition of RSNOs (e.g., *S*‐nitrosoglutathione (GSNO)) in the presence of thiols, a process that mimics the activity of the enzyme glutathione peroxidase (GPx).^[^ ^23^, ^24^ ^]^ Studies have shown that GSNO treatment alone does not effectively inhibit platelet activation; however, combining it with selenocystamine and glutathione (GSH) catalytically generates NO, leading to a significant reduction in platelet activation and fibrin formation.^[^ ^25^ ^]^ Seleno compounds also exhibit anti‐inflammatory properties, further enhancing their potential in medical applications.^[^ ^26^, ^27^ ^]^ Despite the benefits of using seleno compounds for stable NO release, current methods for their incorporation in catheters involve complex, multi‐step surface modification processes.^[^ ^28^, ^29^ ^]^ This highlights the need for simple and versatile strategies for seleno‐mediated catalytic NO generation that can be easily integrated into catheters. One promising approach involves using catechol‐ or plant polyphenol‐based coatings, which can functionalize a wide range of surfaces in a simple, one‐step process.^[^ ^25^ ^]^ This type of coating is attractive due to its broad‐spectrum properties, including anti‐inflammatory, antimicrobial, anti‐thrombosis, and antioxidant effects.^[^ ^30^ ^]^ These properties enable catheter surfaces to achieve stable NO release and simultaneously reduce inflammation, providing a dual‐action solution without the need for complex modification techniques.

**Scheme 1:**
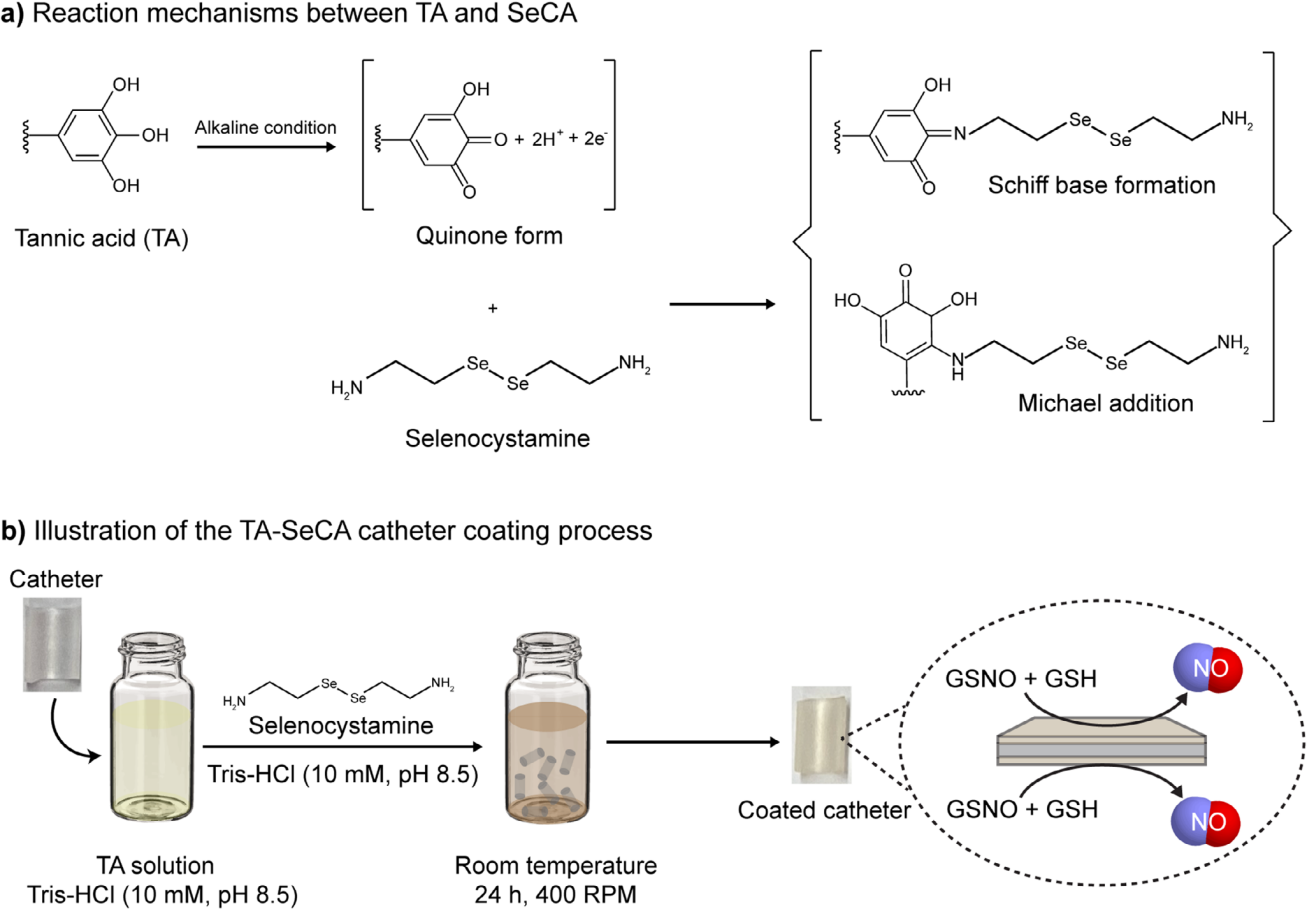
a) Reaction mechanism between tannic acid (TA) and selenocystamine (SeCA). b) Schematic illustration of a TA‐SeCA based coating on a medical‐grade catheter for therapeutic NO generation.

Inspired by this, we developed a novel coating strategy for catheters using selenocystamine (SeCA) and tannic acid (TA). Under mildly alkaline conditions, TA undergoes oxidation to form reactive quinones, which interact with the primary amine groups in SeCA via Schiff base reactions and Michael addition (**Scheme** **1**a).^[^ ^31^, ^32^ ^]^ The interaction between phenolic and amine groups enables a simple, one‐pot method to generate a NO‐releasing coating on catheters (Scheme 1b). The resulting phenol‐amine‐coated materials demonstrated stable and tunable NO release in the presence of GSNO and GSH, offering precise NO delivery. Additionally, under dynamic flow conditions simulating intravenous infusion, the TA‐SeCA coated catheter exhibited rapid, stable, and consistent NO generation over repeated cycles. Stability tests confirmed the coating's durability and effectiveness under various sterilization conditions. This material‐driven approach provides a simple yet effective strategy for NO delivery to reduce catheter‐related complications. In addition to its biocompatibility with human coronary artery smooth muscle cells (HCASMCs) and excellent hemocompatibility, the TA‐SeCA coating significantly reduced the release of pro‐inflammatory cytokines, including TNF‐α and IL‐6, in RAW 264.7 macrophages upon lipopolysaccharide (LPS) stimulation. These findings further highlight the promise of the TA‐SeCA coating for maintaining vascular function, reducing inflammation, and supporting long‐term catheter safety.

## 2 Results and Discussion

### 2.1 Surface Characterization of Coated Catheters

**Figure 1:**
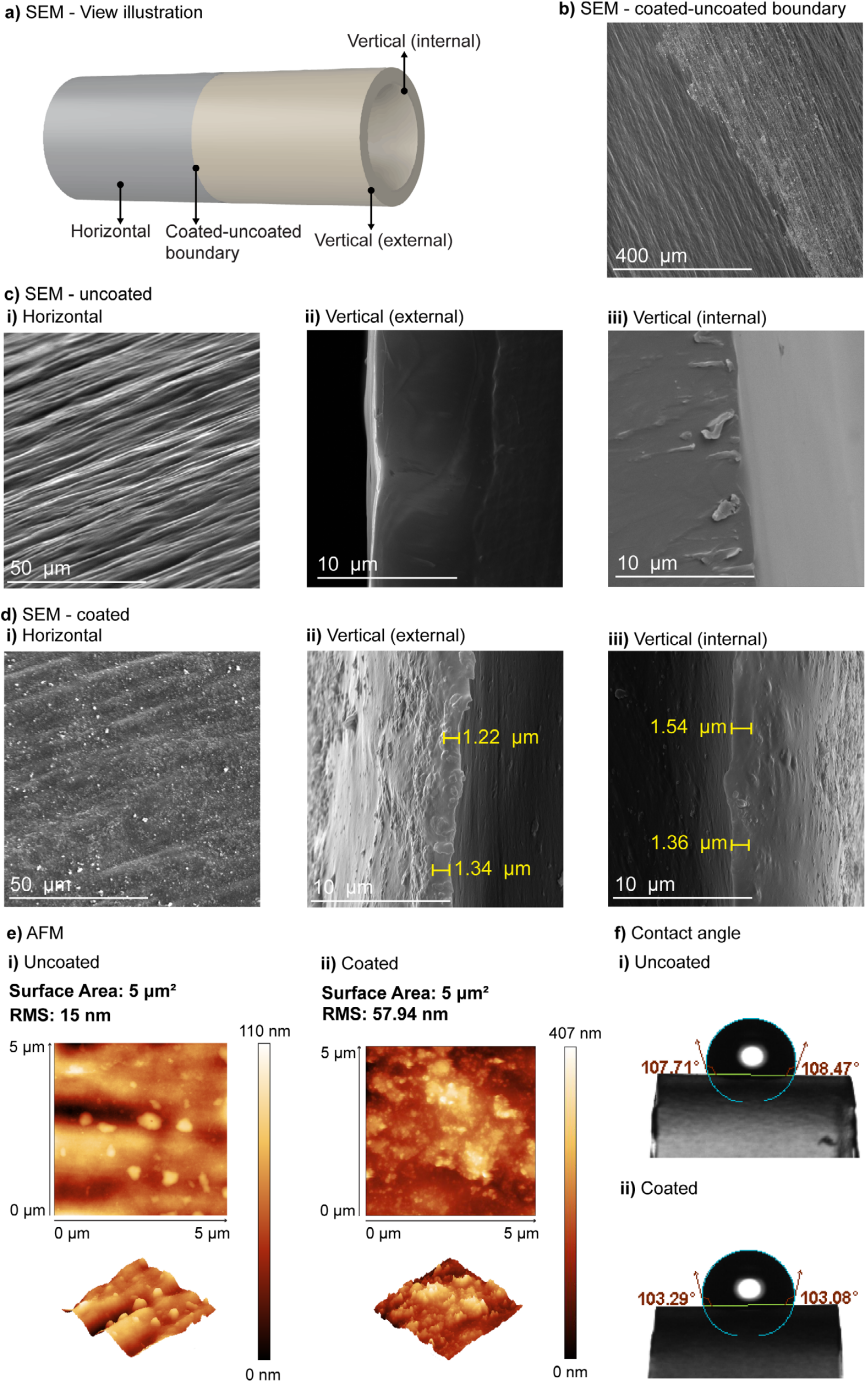
Surface characterization of uncoated and coated catheter segments. a) Schematic illustration indicating the regions of the tubular catheter segment selected for SEM imaging. b) SEM image of the catheter segments coated‐uncoated boundary. SEM images of c) uncoated and d) coated catheter segments from i) horizontal, ii) vertical (external), and iii) vertical (internal) orientations. e) AFM images of i) uncoated and ii) coated catheter segments. f) Contact angle measurements of i) uncoated and ii) coated catheter segments.

To understand the impact of coating on the physical properties of catheter materials, a comprehensive analysis of uncoated and coated catheter segments was performed to assess surface morphology and properties. Tubular catheter segments (5 mm in length, 3 mm in diameter) were immersed in a solution containing TA and SeCA at various molar ratios (1:0.1, 1:0.5, 1:1, 1:2, 1:4, and 1:8) for 24 h at room temperature. The surface morphology of the catheter segments was then examined using SEM (**Figure** **1**a–d), with the various regions selected for imaging outlined in Figure 1a. To facilitate a precise and easy comparison between coated and uncoated surfaces, the coating was applied to only half of each tubular catheter segment, allowing for direct side‐by‐side evaluation. Figure 1b shows the boundary between coated and uncoated regions of the catheter segment, clearly highlighting the differences between the two surfaces. The uncoated region exhibited a smooth morphology (Figure 1c), indicating the absence of surface modification. On the other hand, the coated region displayed increased surface roughness on both the outer and inner surfaces (Figure 1d), suggesting the successful application of the coating, with an approximate thickness of 1.34 µm. This increase in surface roughness was confirmed using AFM, which showed a 3.8× increase in root mean square of the surface (Figure 1e). Despite these changes in surface roughness, contact angle measurements (Figure 1f) demonstrated that the hydrophobic properties of both uncoated and coated catheter segments remained largely unchanged. The contact angle measurements for the different coating ratios showed minimal variations, with mean values ranging from 100.33 to 106.63° (Table S1, Supporting Information). This suggests that while the coating increases the surface roughness, it does not significantly alter the inherent hydrophobicity of the catheter segments. Furthermore, EDS data showed a uniform distribution of elements across the surface (Figure S1, Supporting Information), indicating a homogenous coating without irregularities.

**Figure 2:**
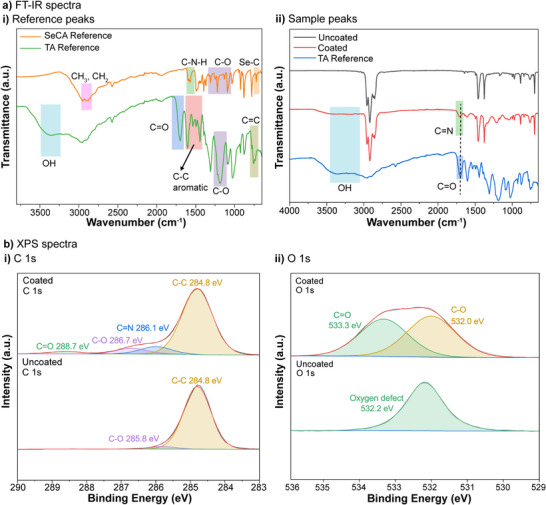
a) FT‐IR spectra of i) pure TA or SeCA and ii) uncoated catheter segments, TA‐SeCA coated catheter segments, or pure TA. b) XPS spectra of uncoated and Ta‐SeCA coated catheter segments: i) C 1s and ii) O 1s.

Next, Fourier transform infrared spectroscopy (FT‐IR) analysis was conducted to confirm the successful reaction between TA and SeCA on the coated catheter segments (**Figure** **2**a). The FT‐IR spectra were obtained for pure SeCA and TA (Figure 2ai), which were consistent with previously reported data.^[^ ^33^, ^34^ ^]^ The TA spectrum displayed a broad absorption band ≈3358 cm^−1^, attributed to ‐OH groups, and characteristic peaks at 1700 cm^−1^ corresponding to C = O stretching. Additional peaks were observed between 1603 to 1534 cm^−1^ and at 1300 to 1000 cm^−1^, indicating C‐C and C‐O stretches, respectively. A peak at 759 cm^−1^ indicated the presence of C = C bonds in a trisubstituted benzene ring. For SeCA, peaks at 2954 and 2882 cm^−1^ were attributed to CH_3_ and CH_2_ groups, while a peak at 723 cm^−1^ corresponded to Se‐C bonds. Peaks between 1310 and 1095 cm^−1^ were associated with C‐O groups, and a possible C‐N‐H bending vibration was noted at 1570 cm^−1^. The FT‐IR spectra of uncoated catheter segments, catheter segments coated with only SeCA or TA, and catheter segments coated with various ratios of TA:SeCA were then obtained (Figure S2, Supporting Information). Notably, compared to the uncoated catheter segments, the spectra of the catheter segments coated with only SeCA or only TA showed no new peaks, suggesting that a stable coating cannot be formed using either compound alone (Figure S2a, Supporting Information). Figure 2aii shows the formation of C = N bonds on the TA‐SeCA coated catheter segments, indicated by the peak at 1695 cm^−1^, which suggests that a Schiff base reaction occurred between TA and SeCA, consistent with previous findings.^[^ ^25^ ^]^ The presence of these C = N bonds confirms that the chemical interaction between TA and SeCA resulted in a stable coating on the catheter segments. Moreover, the increasing intensity of the C = N peak with higher SeCA concentrations (Figure S2b, Supporting Information) suggests that a greater number of imine bonds are formed as more SeCA is incorporated.

To further characterize the chemical composition and surface electronic state of the uncoated and coated catheter segments, XPS was employed (Figures S3–S9, Supporting Information). The XPS spectra confirmed the presence of nitrogen (N) and selenium (Se) on all coated catheter segments, indicating the successful incorporation of TA and SeCA. As shown in Table S2 (Supporting Information), the atomic percentage of Se increased with higher SeCA concentrations but decreased at a ratio of 1:8. This decrease implies an optimal ratio for the incorporation of SeCA, beyond which the efficiency of Se binding decreases. Additionally, as shown in Figure 2b, the new peak at the binding energy of 286.1 eV of carbon (C) element in the coated catheter segments corresponds to the C = N bond.^[^ ^35^ ^]^ This finding further validates the FT‐IR results, supporting the occurrence of a Schiff base reaction between TA and SeCA. Together, these characterization techniques confirm that the TA‐SeCA coatings were successfully applied to the catheter surfaces.

### 2.2 NO Generation

**Figure 3:**
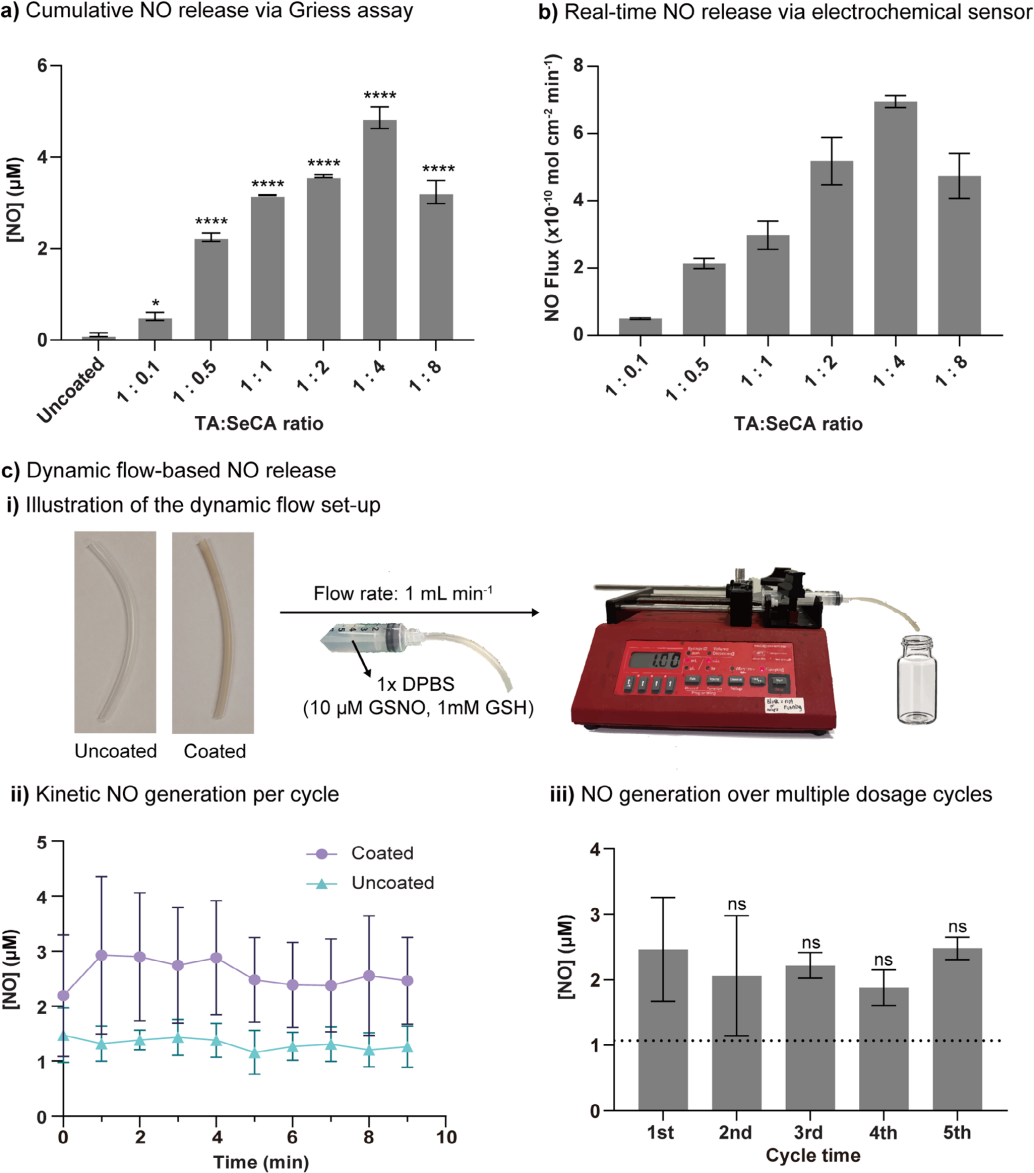
a) Cumulative NO generation after 30 min measured via Griess assay (absorbance at 546 nm), and b) maximum NO flux measured using an electrochemical sensor for GSNO (10 µM) in the presence of GSH (1 mM) in HEPES buffer (pH 7.4) at 37 °C. Measurements were taken for an uncoated catheter segment and catheter segments with TA:SeCA molar ratios from 1:0.1 to 1:8. c) Dynamic, flow‐based NO generation using a perfusion system: i) schematic illustration of the experimental setup with a 10 cm catheter segment connected to a syringe and pump for continuous injection; ii) representative kinetic NO release profile during a single 10 min injection cycle using uncoated and coated catheter segments at a flow rate of 1 mL min^−1^ in DPBS buffer containing GSNO (10 µM) in the presence of GSH (1 mM), followed by iii) cumulative NO generation across five dosage cycles. The dashed line represents the uncoated control in the same conditions. Statistical significance relative to control tests was calculated using one‐way ANOVA, ns = not significant, *p < 0.1, ****p < 0.0001. n = 3; error bars represent standard deviation.

Next, TA‐SeCA coated tubular catheter segments were evaluated for their ability to generate NO when exposed to the NO donor GSNO in the presence of GSH. GSH was included as a reducing agent because the selenium‐based compounds in the coating require GSH to facilitate the NO generation from GSNO.^[^ ^36^, ^37^ ^]^ Uncoated or coated catheter segments were incubated with 10 µM GSNO and 1 mM GSH in HEPES buffer at 37 °C for varying time intervals (0, 15, 30, 40, and 60 min). The concentrations of GSNO (10 µM) and GSH (1 mM) used were based on the reported levels in healthy human samples.^[^ ^38^ ^]^ Following incubation, a Griess assay was performed on the supernatant to quantify the amount of NO generated (Figure S10, Supporting Information). Each experiment used a single 5‐mm catheter segment. The results showed that in the absence of GSH, all coated catheters exhibited minimal NO release over time (Figure S10a, Supporting Information). However, when GSH was present, a significant increase in NO release was observed across all coated catheters (Figure S10b, Supporting Information), indicating that GSH is essential for enhancing NO generation from the coated surfaces. As the maximum NO generation for all coating molar ratios was reached at 30 min, this time point was chosen to evaluate the NO generation capacity of the different coating molar ratios (**Figure** **3**a). A linear increase in NO generation with increasing molar ratio of SeCA relative to TA was observed, with peak cumulative NO generation (4.86 µM) reached at a TA:SeCA ratio of 1:4. This suggests that higher concentrations of SeCA enhance the decomposition of GSNO in the presence of GSH. However, when using a 1:8 coating ratio, a decrease in NO release to 3.23 µM was observed. XPS analysis, as shown in Table S2 (Supporting Information), indicates that the atomic percentage of Se increased from 1.09% at a 1:2 ratio to 1.16% at 1:4, but decreased to 1.02% at a 1:8 ratio. This suggests that beyond a 1:4 ratio, excess SeCA may reduce the availability of active catalytic sites, aligning with the observed reduction in NO generation.

Although the Griess assay is a widely used technique for detecting NO, it has limitations, such as its indirect measurement of nitrite and susceptibility to interferents.^[^ ^39^ ^]^ To overcome these limitations and provide real‐time monitoring, an electrochemical sensor was employed to further validate the findings. Coated catheter segments with different coating molar ratios of TA:SeCA were placed in separate glass vials containing HEPES buffer. These vials were incubated at 37 °C and covered with aluminum foil to protect them from light. Following the addition of 10 µM GSNO and 1 mM GSH in HEPES buffer, the real‐time NO generation was monitored (Figure S11, Supporting Information). The maximum NO flux observed for each coating molar ratio is shown in Figure 3b. Consistent with the data obtained from the Griess assay (Figure 3a), a linear increase in NO flux with increasing molar ratio of SeCA relative to TA was observed. As before, a peak was reached at a TA:SeCA ratio of 1:4 at 7 × 10^−10^ mol cm^−2^ min^−1^, validating that the 1:4 ratio provides the most efficient NO generation. These results show that the NO generation can be fine‐tuned by adjusting the coating molar ratio, allowing for controlled release rates to meet different therapeutic needs.

To further evaluate the clinical potential of the NO‐generating TA‐SeCA coating, we assessed its performance under dynamic flow conditions designed to simulate the physiological environment experienced by vascular catheters. Clinically, catheters are commonly utilized for intravenous (IV) infusion, central venous access, and peritoneal dialysis. Among these applications, IV infusion closely mirrors steady‐state, low‐shear conditions suitable for modeling NO release. Clinical guidelines typically suggest adult IV infusion rates ranging from 0.5 to 1.5 mL min^−1^; thus, a flow rate of 1 mL min^−1^ was selected for our experiments to closely mimic clinically relevant perfusion conditions.^[^ ^40^ ^]^ In this experiment, a 10 cm segment of either uncoated or TA‐SeCA coated catheter tubing (prepared at a 1:4 molar ratio) was connected to a 10 mL syringe containing a solution of GSNO (10 µM) and GSH (1 mM) in DPBS to better mimic physiological fluid conditions (Figure 3ci). The solution was perfused through the tubing at a rate of 1 mL min^−1^ using a syringe pump. Effluent samples were collected at 1‐min intervals over a total duration of 10 min for NO quantification using the Griess assay (Figure 3cii). Results indicated rapid, stable and consistent NO generation kinetics from the TA‐SeCA coated catheter across the entire 10‐min cycle. In contrast, the uncoated catheter displayed minimal NO generation under identical conditions. Additionally, to assess the durability and consistency of the NO‐generating capacity of the coating, the injection cycle was repeated five consecutive times (Figure 3ciii). Across all cycles, the TA‐SeCA coated catheter demonstrated stable and consistent NO release without significant variation in NO generation between cycles. These results showed the robustness and reliability of the TA‐SeCA coating under dynamic flow conditions, highlighting its promising translational potential for application in peripherally inserted central catheters (PICCs) or other long‐term intravascular devices commonly utilized in chemotherapy and chronic infusion therapies.

### 2.3 Antioxidant Properties

**Figure 4:**
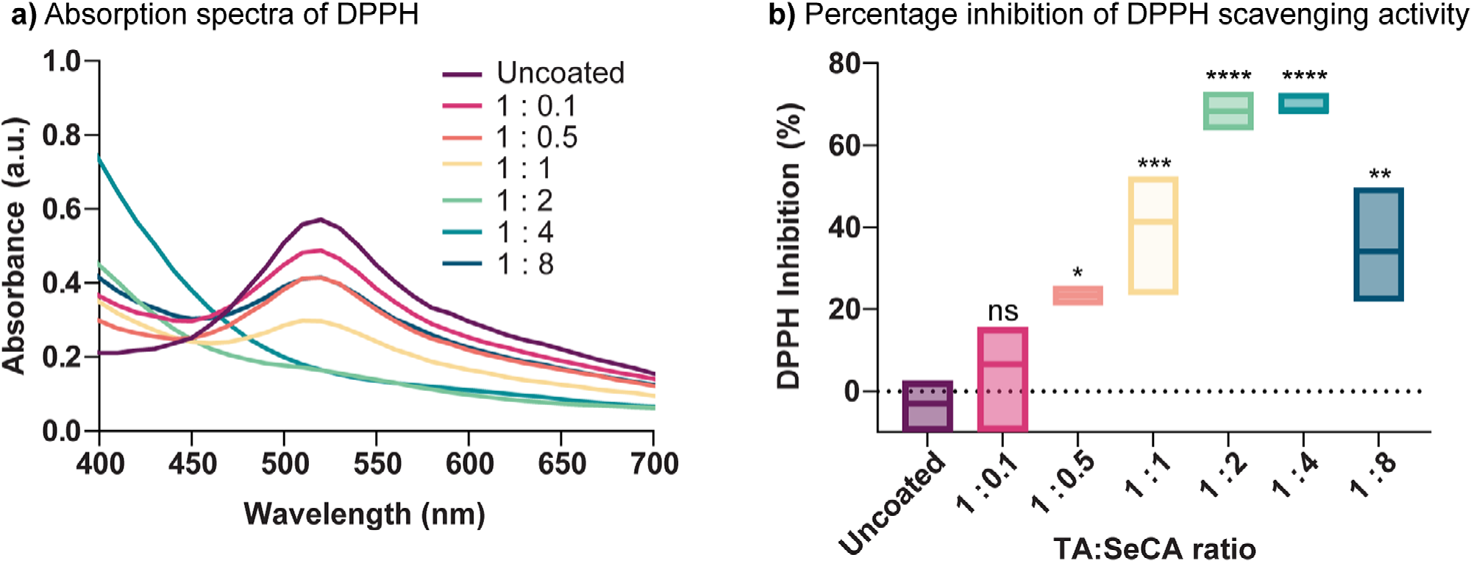
a) UV–vis spectra of 1 mM DPPH radical (DPPH•) and b) percentage inhibition of DPPH scavenging activity for uncoated catheter segments and TA:SeCA coated at molar ratios from 1:0.1 to 1:8. Statistical significance relative to control tests was calculated using one‐way ANOVA, ns = not significant, *p < 0.1, **p < 0.01, ****p < 0.0001. n = 3; error bars represent standard deviation.

Given the known antioxidant capabilities of TA^[^ ^41^ ^]^ and seleno compounds,^[^ ^42^ ^]^ we evaluate the radical scavenging performance of TA‐SeCA coatings applied to catheter surfaces at different molar ratios using a DPPH assay. In this assay, a stable free radical with a deep violet color, showing maximum absorbance at ≈517 nm, is utilized. The presence of an antioxidant leads to electron or hydrogen atom donation, neutralizing the DPPH radical and causing a color change from violet to yellow.^[^ ^43^ ^]^ Briefly, 1 mM DPPH solution dissolved in ethanol was incubated with uncoated or coated catheter segments at various coating molar ratios for 12 h. After incubation, the supernatant was collected, and its spectrum was measured from 400 nm to 700 nm (**Figure** **4**a). The spectrum of the uncoated catheter displayed a distinct peak ≈517 nm, corresponding to the presence of DPPH free radicals. However, as the coating molar ratio increases, this peak at 517 nm decreases, indicating a reduction in the purple color intensity of the DPPH solution. This decrease suggests that higher coating ratios are more effective at scavenging free radicals, thereby demonstrating improved antioxidant properties. Consequently, the percentage of DPPH scavenging inhibition was calculated and is shown in Figure 4b, where the 1:2 and 1:4 molar ratios exhibited the highest levels of DPPH inhibition (≈70%), indicating these ratios are optimal for maximizing antioxidant capacity. This suggests that SeCA, tuned through varying molar ratios, plays a significant role in enhancing the antioxidant activity of these coatings.

### 2.4 Coating Recyclability and Stability of NO Generation

**Figure 5:**
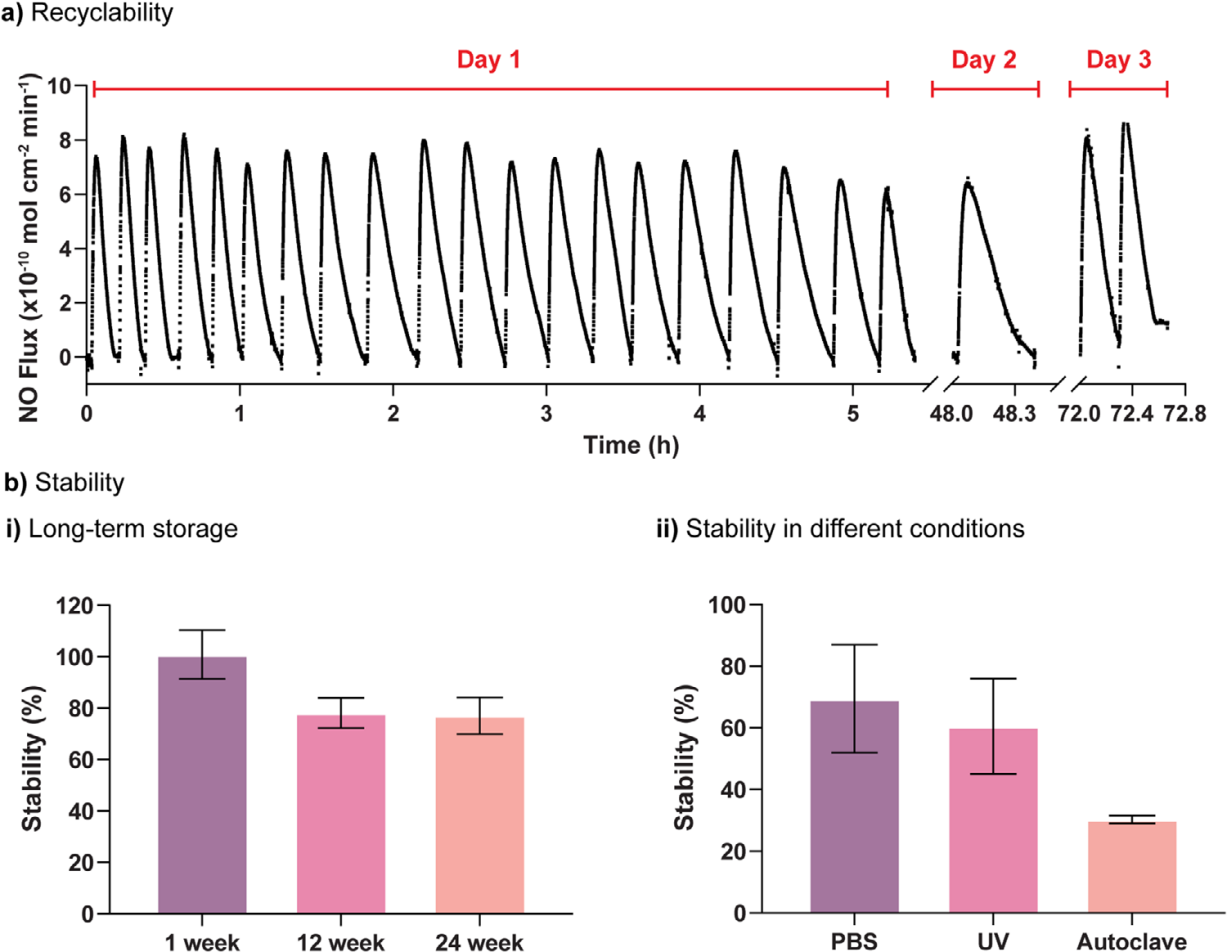
Recyclability and stability of TA‐SeCA (1:4) coated catheter segments. a) Representative NO flux profile over 72 h at 37 °C, showing multiple GSNO additions on day 1, followed by one addition on day 2 and two additions on day 3. b) Calculated stability (%) under i) long‐term storage at room temperature (1, 12, and 24 weeks), and ii) different conditions. All experiments were exposed to GSNO (10 µM) in the presence of GSH (1 mM) in HEPES or PBS buffer (pH 7.4) at 37 °C. n = 3; error bars represent standard deviation.

Following evaluation of the catheter's NO‐generating and antioxidant properties, a coating ratio of 1:4 was identified as the most effective formulation. As such, this ratio was used for further studies that assess recyclability and stability. First, when evaluating long‐term sustained delivery, a critical factor is the ability to sustain NO after multiple uses. To assess this, a coated catheter segment was incubated with 1 mM GSH in HEPES buffer, and 10 µM GSNO was added repeatedly over 72 h. An electrochemical sensor was then used to record the real‐time NO generation (**Figure** **5**a). The coated catheter segments showed sustained NO generation at 7–8 × 10^−10^ mol cm^−2^ min^−1^ over this 3‐day period, suggesting excellent stability over repeated use. However, it should be noted that the time required to generate NO from 10 µM GSNO increased over time from ≈10 to 20 min by days 2 and 3. This suggests a decline in the catalytic activity of the coating and is likely due to the loss or degradation of the coating over time. To explore this further, FT‐IR spectra of the coated catheter segments were recorded daily. As shown in Figure S12 (Supporting Information), there is a decrease in the C = N peak from day 1 to day 3, indicating potential structural degradation of the coating. These findings highlight the importance of further optimizing the coating's stability for sustained catalytic performance. However, the U.S. CDC guidelines recommend replacement of peripheral intravenous catheters (PIVC) every 72 to 96 h.^[^ ^44^ ^]^ Therefore, considering that catheters are typically used for less than three days, this modest decline in performance is unlikely to pose a significant clinical concern.

The stability of coated catheter segments was then examined, as potential applications may require long‐term storage. Therefore, the effect of storage time on NO generation was investigated. Briefly, coated catheter segments were stored for 1, 12, and 24 weeks at room temperature, and a real‐time NO measurement was carried out via a free‐radical analyzer to determine the NO flux profile (Figure S13a, Supporting Information). No decrease in catalytic activity was observed over a 1‐week storage period (Figure 5bi). However, there was a 20% loss of catalytic activity after both 12 and 24 weeks of storage. In addition to assessing long‐term stability, it is crucial to evaluate the coating's performance under conditions that medical devices may encounter in clinical settings following the Australian Standards AS/NZS 4815:2006. To this end, the NO flux profile was examined under various conditions, including incubation in PBS, autoclaving (121 °C, 103 kPa, 15 min), and UV exposure for 30 min (Figure S13b, Supporting Information). As shown in Figure 5bii, the catalytic activity remained above 70% when tested in PBS and above 60% after UV light exposure. However, after autoclaving, the catalytic activity decreased to 30%, indicating substantial loss of functionality under these harsher sterilization conditions. The 1:4 coating ratio demonstrates promise for sustained NO generation under certain conditions. However, the observed decline in catalytic activity over time and after specific sterilization procedures highlights the need for further optimization. Future research should focus on improving the coating formulation to maintain consistent performance and functionality across diverse and challenging environments to ensure viability for long‐term clinical use.

### 2.5 Biocompatibility, Endogenous NO Generation, and Hemocompatibility

**Figure 6:**
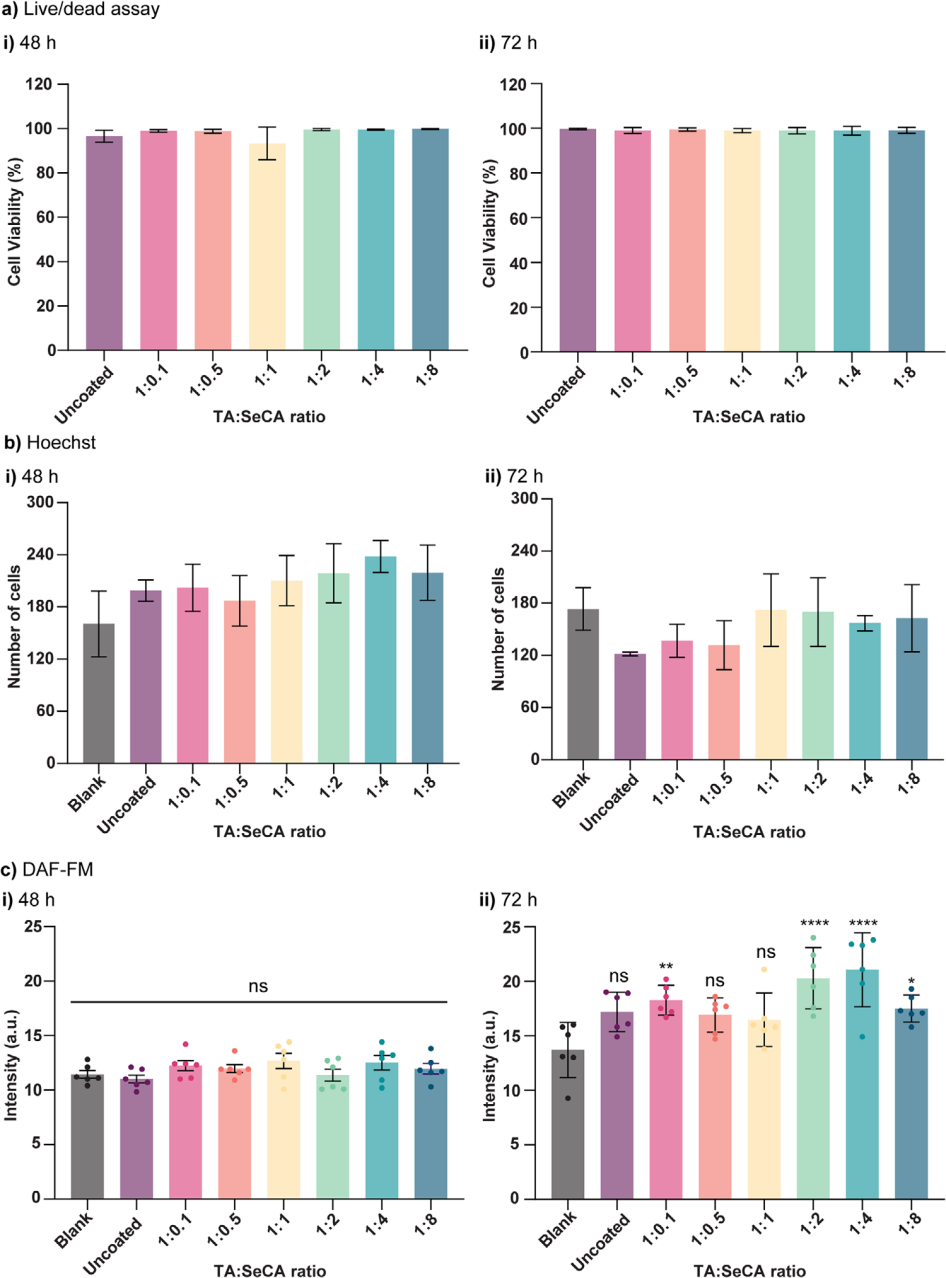
a) HCASMCs viability, b) number of cells, and c) endogenous NO generation after incubation with uncoated and coated catheter segments compared to the blank group, measured using the Live/Dead assay, Hoechst staining, and DAF‐FM diacetate, respectively, at i) 48 h and ii) 72 h. Statistical significance relative to control tests was calculated using one‐way ANOVA, ns = not significant, *p < 0.1, **p < 0.01, ****p < 0.0001. n = 6; error bars represent standard deviation.

To evaluate the compatibility of TA‐SeCA coated catheter segments with vascular environments, both cellular and blood‐contact assessments were performed. Human coronary artery smooth muscle cells (HCASMCs) were selected to evaluate the biocompatibility of the coated catheter segments, as they are representative of the cells that would interact with catheters in clinical use and play a role in maintaining vessel wall integrity.^[^ ^45^ ^]^ Ensuring good biocompatibility with HCASMCs helps confirm that the coated materials do not trigger adverse cellular responses, such as inflammation or cytotoxicity. In this study, both uncoated and coated catheter segments were exposed to HCASMCs for 48 and 72 h, and cell viability was assessed using a Live/Dead assay. As depicted in Figure S14 (Supporting Information), cells incubated with the coated catheter segments were stained with calcein‐AM and ethidium homodimer‐1 (EthD‐1) for live and dead cell markers, respectively. HCASMCs exhibited regular cell morphology, and uptake of calcein‐AM with minimum EthD‐1 fluorescence across all groups suggests no cytotoxicity. Quantitative analysis of the Live/Dead assay confirmed that cell viability remained above 96% across various coating ratios (1:0.1 to 1:8) (**Figure** **6**a).

In addition, the cell proliferation rate was assessed over 48 and 72 h with nucleic acid counterstain Hoechst for cell number (Figures S15 and S16, Supporting Information). Cell proliferation analysis is conducted to eliminate the risk of intimal hyperplasia, a condition characterized by excessive smooth muscle cells (SMCs) proliferation within the vascular wall,^[^ ^46^ ^]^ which can narrow the vessel lumen and impede blood flow. Over the past two decades, many drugs for intimal hyperplasia have focused on suppressing local immune responses and inhibiting abnormal SMC proliferation.^[^ ^47^, ^48^ ^]^ Figure 6b shows no significant increase in HCASMCs numbers compared to the blank control group across all samples at both time points. This suggests that the coated catheter segments may prevent excessive proliferation and help control HCASMCs growth over time. These results indicate that the coated catheter segments align with the desired profile for intravascular devices, indicating they can safely interact with vascular tissues. Future studies would be beneficial to investigate endothelial cell proliferation to ensure that the coated catheter segments support endothelial cell growth.

Next, we evaluated whether the TA‐SeCA coating could enhance endogenous NO generation from vascular cells, relying exclusively on endogenous GSNO without external supplementation, to evaluate the therapeutic potential. HCASMCs were incubated with intracellular NO fluorescent probe DAF‐FM diacetate to evaluate endogenous NO generation between the coated catheter and blank control. Notably, the blank group also exhibited NO expression, as detected by DAF‐FM (Figures S15 and S16, Supporting Information), indicating the presence of NO within smooth muscle cells without the addition of an exogenous source. This observation aligns with previous studies, which suggest that human vascular smooth muscle cells in culture are capable of producing and releasing biologically active NO from L‐arginine or via nitric oxide synthase (NOS).^[^ ^49^, ^50^ ^]^ The mean DAF‐FM fluorescence intensity for each group was analyzed to assess NO production. As shown in Figure 6ci, there was no significant difference in NO generation at 48 h between uncoated and coated catheter segments at various molar ratios. However, by 72 h, the catheter segments coated at ratios of 1:2 and 1:4 demonstrated a significant upregulation in fluorescence intensity compared to the blank control (Figure 6cii). This observation after 72 h suggests that the coated catheter segments with specific molar ratios (1:2 and 1:4) upregulate NO in a time‐dependent manner. The lack of significant NO production at 48 h implies that the initial interaction between the coating and the cells does not immediately stimulate NO synthesis. The role of NO in SMCs is crucial for regulating vascular tone and function. Previous studies have shown that NOS maintain the contractile phenotype of SMCs,^[^ ^51^ ^]^ suggesting that NO plays a regulatory role in controlling contractile force, contributing to vasodilation and maintaining vascular homeostasis.^[^ ^52^ ^]^ Therefore, the increased NO production observed at 72 h with coated catheter segments at the 1:2 and 1:4 molar ratios could have therapeutic potential. This is particularly relevant for conditions like thrombosis, where NO‐mediated vasodilation and the prevention of platelet aggregation offer significant benefits. Further mechanistic studies aim to focus more on the cellular pathways involved and to validate the observed effects in more complex models.

**Figure 7:**
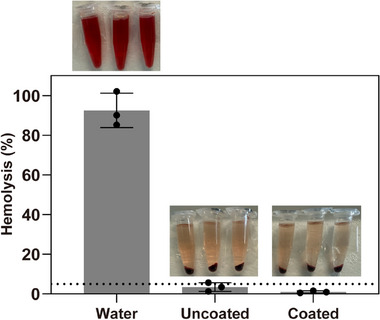
Hemolysis percentage of RBCs treated with uncoated and coated catheter segments for 2 h. RBCs treated with water served as the positive control, and DPBS‐treated RBCs served as the negative control. The dashed line indicates the 5% hemolysis threshold. n = 3; error bars represent standard deviation.

Drawing blood through IV catheters is also associated with potential risk of hemolysis.^[^ ^53^, ^54^ ^]^ To directly evaluate hemocompatibility, a hemolysis analysis was performed using murine red blood cells (RBCs) obtained by cardiac puncture from humanely anesthetized mice. Both uncoated and coated catheter segments (1:4 ratio) were incubated with a 2% (v/v) RBCs suspension for 2 h. As shown in **Figure** **7**, the coated segments induced <1% cell lysis and well below the 5% threshold defined by the ISO 10 993/4 standard,^[^ ^55^ ^]^ showing the nonhemolytic nature and excellent hemocompatibility. While current studies focused on in vitro characterization, the observed biocompatibility, hemocompatibility, and NO‐modulating effects of the TA‐SeCA coating offer strong justification for future in vivo validation in animal models and support its potential application in blood‐contacting medical devices. These studies collectively support the potential of the coating system for intravascular applications where safety and hemocompatibility are important.

### 2.6 NO‐Regulated Inflammatory Responses

**Figure 8:**
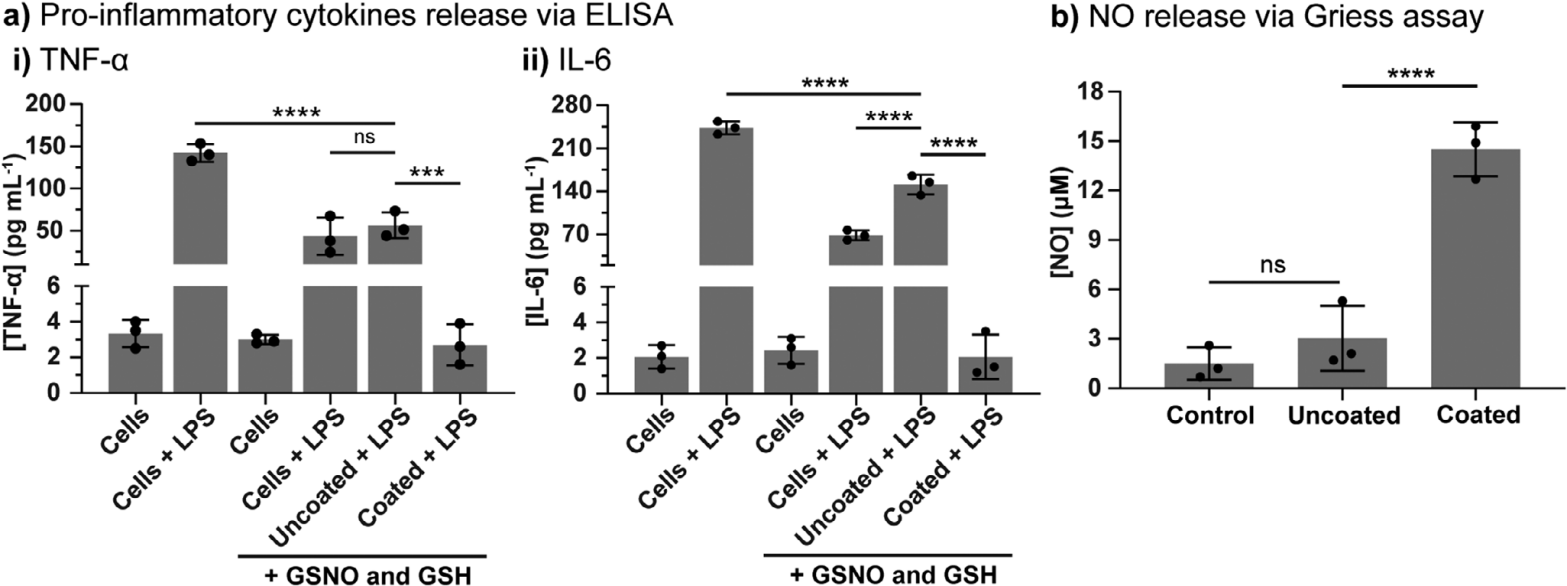
Effects of TA‐SeCA coated catheter segments on LPS‐induced inflammatory responses in RAW264.7 macrophages. a) Pro‐inflammatory cytokines i) TNF‐α and ii) IL‐6 release measured via ELISA. c) Cumulative NO release from samples in LPS‐induced RAW264.7 macrophages measured via Griess assay. Statistical significance relative to control tests was calculated using one‐way ANOVA, ns = not significant, ***p < 0.001, ****p < 0.0001. n = 3; error bars represent standard deviation. TNF‐α, tumor necrosis factor‐α; IL‐6, interleukin‐6.

The effects of coated catheter segments on LPS‐induced inflammatory responses were evaluated using RAW 264.7 macrophages, a widely used myeloid cell line for studying immune responses.^[^ ^56^, ^57^ ^]^ Upon LPS stimulation, RAW 264.7 cells display upregulated NO production through iNOS and activate the release of key pro‐inflammatory cytokines, including TNF‐α, IFN‐γ, IL‐1β, and IL‐6.^[^ ^56^, ^58^ ^]^ By studying the pro‐inflammatory responses in RAW 264.7 macrophages, we can gain valuable insights into how coated catheter segments influence the immune response, particularly in the regulation of NO and cytokine production. Therefore, the effect of TA:SeCA coated catheter segments on LPS‐induced TNF‐α and IL‐6 release was evaluated in RAW 264.7 macrophages (**Figure** **8**a,b). In the absence of GSNO and GSH, LPS stimulation led to a significant 43‐fold increase in TNF‐α secretion compared to the control group without LPS stimulation (Figure 8ai). When RAW 264.7 macrophages were supplemented with GSNO (30 µM) and GSH (90 µM), with or without uncoated catheter segments, TNF‐α levels also showed a 19‐fold and 14‐fold increase, respectively. This suggests that while the addition of GSNO and GSH reduced TNF‐α levels relative to the LPS‐only control group, the levels remained relatively high, at ≈43 pg mL^−1^ without a catheter segment and 56 pg mL^−1^ with an uncoated catheter segment, respectively. In contrast, the combination of GSNO and GSH with the TA:SeCA coated catheter segments significantly lowered TNF‐α levels to ≈3 pg mL^−1^, comparable to the control group without LPS stimulation. Similarly, in the absence of GSNO and GSH, LPS stimulation led to a significant 118‐fold increase in IL‐6 secretion compared to the control group without LPS stimulation (Figure 8aii). When RAW 264.7 macrophages were exposed to GSNO and GSH, with or without uncoated catheter segments, IL‐6 levels were elevated, showing a 62‐fold and 28‐fold increase, respectively. In contrast, the combination of GSNO and GSH with TA‐SeCA coated catheter segments significantly reduced IL‐6 levels, bringing them down to ≈2 pg mL^−1^, comparable to the control group without LPS stimulation. Both TNF‐α and IL‐6 levels were elevated when macrophages were exposed to LPS in the absence of NO donors or coatings, indicating a robust inflammatory response. The substantial increase in pro‐inflammatory cytokine expression observed with uncoated catheters showed signs of inflammatory response, highlighting the importance of incorporating anti‐inflammatory coatings to mitigate these effects. However, the application of TA‐SeCA coated segments, combined with GSNO and GSH, effectively reduced the levels of these key pro‐inflammatory cytokines to near‐baseline values observed in non‐LPS stimulated control cells. This significant reduction in cytokine secretion suggests that TA:SeCA coatings, in combination with GSNO and GSH, provide a synergistic anti‐inflammatory effect. To further investigate the underlying mechanism, cumulative NO production was measured in the supernatants of macrophages treated with various formulations following LPS stimulation and the addition of GSNO and GSH, using the Griess assay. As depicted in Figure 8b, NO generation was significantly increased with the TA:SeCA coated catheter segments compared to the uncoated or control cell groups. This finding further supports the hypothesis that the reduction in pro‐inflammatory cytokines is likely mediated through modulation of NO signaling pathways and suppression of pro‐inflammatory cytokine production. Taken together, these results show that TA:SeCA coated catheter segments, in combination with GSNO and GSH, can significantly reduce inflammatory responses in macrophages, making them a promising strategy for anti‐inflammatory treatment of catheter implants.

## 3 Conclusion

Catheters are essential in modern medical care but are often associated with complications that affect both device performance and patient outcomes. While current solutions like surface coatings and drug‐eluting systems offer some protection, they are often hindered by limited drug reservoirs, complex fabrication, and poor long‐term stability. Similarly, methods for localized drug synthesis, though innovative, are restricted by the instability of enzyme‐based systems. In this study, we developed a simpler, scalable alternative by utilizing the inherent properties of TA and SeCA to create a phenol‐amine‐based coating that catalytically generates NO. By optimizing the TA‐SeCA molar ratio, we achieved stable and tunable NO release, with the 1:4 ratio being the most effective for generating therapeutic levels of NO. Our findings demonstrate that this coating not only shows excellent biocompatibility with HCASMCs but also significantly reduces LPS‐induced inflammatory responses in RAW 264.7 macrophages. Additionally, under dynamic flow conditions simulating clinical usage, the coating maintained consistent NO generation, further supporting its potential in clinical settings. The combination of TA:SeCA coated catheter segments with GSNO and GSH resulted in substantial reductions in key pro‐inflammatory cytokines, TNF‐α and IL‐6, highlighting its potential to mitigate inflammation, infection, and thrombosis. Additionally, the coating demonstrated robust stability, maintaining catalytic performance across multiple cycles and under UV sterilization conditions. This material‐driven approach simplifies NO delivery and offers a reliable, scalable, and stable solution to mitigate catheter‐related complications. By integrating anti‐inflammatory, hemocompatible, and NO‐generating capabilities, the TA:SeCA coating presents a versatile and practical strategy to improve the biocompatibility and long‐term safety of catheter implants in clinical settings.

## 4 Experimental Section

### Materials

Medical grade polyvinyl chloride (PVC) tubes (diameter 3 mm) were purchased from Shandong Wego Group Medical Polymer Co., Limited. Tannic acid (TA), selenocystamine dihydrochloride (SeCA), Tris hydrochloride (Tris‐HCl), 4‐(2‐hydroxyethyl)piperazine‐1‐ethanesulfonic acid (HEPES), Griess reagent (modified), L‐glutathione reduced (GSH), ethanol, and 2,2‐diphenyl‐1‐picrylhydrazyl (DPPH) were purchased from Sigma–Aldrich. DPBS (14 190 144), Hoechst 33 342 and DAF‐FM diacetate (D23842) were purchased from Thermo Fisher. LIVE/DEAD viability/cytotoxicity kit for mammalian cells was purchased from Invitrogen. GSNO was purchased from MedChemExpress. Human coronary artery smooth muscle cells (HCASMCs) and smooth muscle cell growth medium kit were purchased from Cell Applications. Polylactic acid (PLA) inserts were fabricated using an Ultimaker 3 3D printer (Fused Deposition Modelling). Ultrapure water (18.2 MΩ cm) was provided by arium pro Ultrapure Water Systems (Sartorius). HEPES and Tris‐HCl buffers were prepared by dissolving HEPES or Tris‐HCl powder in ultrapure water and gently stirring until fully dissolved, yielding 10 mM solutions of HEPES (pH 7.4) and Tris‐HCl (pH 8.5). The mice blood was a kind gift from the Bioresources Facility at the University of Melbourne and was obtained from humanely euthanized mice used for training purposes.

### Preparation of TA and SeCA Solutions

All solutions were freshly prepared immediately before use to ensure optimal activity and reproducibility. A TA solution (0.4 mL) was prepared at a concentration of 15 mM in Tris‐HCl buffer. This TA solution was then diluted with an additional volume of Tris‐HCl buffer and thoroughly mixed. Following this, a SeCA solution (15 mM in Tris‐HCl buffer) was added, and the mixture was further mixed to ensure homogeneity. The volumes of SeCA and Tris‐HCl buffer added to achieve the required molar ratios are outlined in **Table** **1**.

**Table 1:** Volumes of TA, SeCA, and Tris‐HCl buffer required for desired TA:SeCA molar ratios.

Ta:SeCA	TA [mL]	SeCA [mL]	Tris‐HCl buffer [mL]
1:0.1	0.40	0.04	3.56
1:0.5	0.40	0.20	3.40
1:1	0.40	0.40	3.20
1:2	0.40	0.80	2.80
1:4	0.40	1.60	2.00
1:8	0.40	3.20	0.40

### Coating of TA‐SeCA on Catheters

PVC catheter tubes were cut to a length of 5 mm and then washed by sonicating (Powersonic 510) in ethanol for 15 min, followed by ultrapure water for another 15 min. After washing, the catheter segments were dried using nitrogen gas and stored for later use. The cleaned catheter segments are referred to as uncoated catheter segments in this study. Coated catheter segments were prepared by immersing the uncoated catheter segments in TA‐SeCA solutions of specific molar ratios, followed by constant vortexing at 400 rpm at room temperature for 24 h using a thermomixer (Eppendorf ThermoMixer C). Following the coating process, the coated catheter segments were sonicated with ultrapure water for 5 min and dried using nitrogen gas.

### Characterization

The morphology and elemental composition of the catheter segments before and after coating were characterized via field emission scanning electron microscopy (FE‐SEM, FEI Nova NanoSEM 450) and energy dispersive spectroscopy (EDS, Bruker SDD‐EDS), respectively. An acceleration voltage of 5 kV was used for FESEM and 15 kV for EDS, both with a spot size of 3. Samples were coated with a 15 nm platinum layer and a 15 nm carbon layer for FESEM and EDS, respectively, using a Leica ACE600 sputter coater.

Atomic force microscopy (AFM) measurements were performed using a Bruker Dimension Icon SPM equipped with a Nanoscope V controller. Peak force tapping mode with the SCANASYST‐AIR probe (Bruker AFM probes) was used on all samples. The scan size was set to 5 µm. The scan rate, peak force, and feedback gain were adjusted accordingly to optimize tracking of the specimen surface, without any significant noise between trace and retrace. The resolution of the image was set to 512 lines per image. Root mean square (RMS) was calculated using Gwyddion software, first by removing polynomial background and then normalizing the height.

Fourier transform infrared (FT‐IR) spectra were recorded using an ATR‐FTIR spectrometer (PerkinElmer Spectrum Two). The spectra were measured from 450 to 4000 cm^−1^ at a resolution of 4 cm^−1^ using 16 accumulated scans.

X‐ray photoelectron spectroscopy (XPS) was conducted using a ESCALAB 250Xi (Thermo Scientific) with binding energies calibrated to the C 1s line at 284.8 eV.

The wetting and adhesion properties of the catheter segments before and after coating were characterized by contact angle measurement using an optical contact angle and surface tension meter (KSV CAM 200 Optical Contact Angle Meter). Ultrapure water droplets (4 µL) were applied to the surface of the substrates, and images were recorded immediately after deposition.

### NO Generation Quantification via Griess Assay

The Griess assay was used to measure the cumulative NO concentrations. TA‐SeCA coated catheter segments were incubated with 1 mL of HEPES buffer containing GSNO (10 µM) and GSH (1 mM) for 0, 15, 30, 40, and 60 min at 37 °C and 200 rpm. Then, 100 µL of the supernatant was incubated with 100 µL of 40 mg mL^−1^ Griess reagent in a 96‐well plate for 15 min protected from light. The absorbance at 546 nm was then measured using a SpectraMax M5 microplate reader. The corresponding NO concentration was calculated via a calibration curve.

### NO Generation Quantification via Electrochemical Sensor

Real‐time NO generation was monitored using a free radical analyzer (TBR4100, World Precision Instruments) equipped with an NO‐sensitive electrode (ISO‐NOP, World Precision Instruments). The NO probe was immersed in a glass vial containing 3.89 mL of HEPES buffer with one TA‐SeCA coated catheter segment. After a stable baseline was reached, 10 µL of GSNO (4 mM in HEPES buffer) and 100 µL of GSH (40 mM in HEPES buffer) were added to achieve a final volume of 4 mL, with final concentrations of 10 µM GSNO and 1 mM GSH. Glass vials were covered with aluminium foil to prevent light exposure and kept at 37 °C on a hot plate with constant stirring (400 rpm) during the NO measurement. Changes in current response were recorded in a real‐time manner and the produced NO was calculated via a calibration curve.

### Dynamic Flow‐based NO Generation

A 10 cm segment of either uncoated or TA‐SeCA coated catheter tubing (1:4 molar ratio) was connected to a 10 mL disposable syringe filled with a 1x DBPS buffer containing 10 µM GSNO and 1 mM GSH. The solution was injected at a constant flow rate of 1 mL min^−1^ through the tubing using a syringe pump. 100 µL eluent was then collected at the outlet in 1 min intervals and incubated with 100 µL of 40 mg mL^−1^ Griess reagent in a 96‐well plate for 15 min protected from light. The absorbance at 546 nm was then measured using a SpectraMax M5 microplate reader. The corresponding NO concentration was calculated via a calibration curve. The procedure was repeated over five consecutive injections using fresh GSNO and GSH solutions to evaluate the stability of NO production across multiple dosing cycles.

### Antioxidant Assay

The antioxidant performance was tested using the DPPH assay. One uncoated or coated catheter segment was immersed in 1 mL of DPPH (1 mM in ethanol) and incubated in a thermomixer for 12 h at 37 °C and 400 rpm. After incubation, 100 µL of the sample supernatant was collected from each Eppendorf tube and transferred to a 96‐well plate. The absorbance of the supernatant was measured using a SpectraMax M5 microplate reader, scanning from 400 to 700 nm. The DPPH inhibition was calculated based on the following equation:

(1) $$DPPH\;inhibition\left(\%\right)=\frac{A_{0}-A_{1}\;}{A_{0}}\times 100$$

where A_0_ is the absorbance of control and A_1_ is the absorbance of reaction mixture, both measured at 517 nm.

### Recyclability of Coated Catheter Segments

The NO‐generating recyclability of the TA‐SeCA coating was evaluated using a free radical analyzer (TBR4100, World Precision Instruments) equipped with an NO‐sensitive electrode (ISO‐NOP, World Precision Instruments). The NO probe was immersed in a glass vial containing 3.89 mL of HEPES buffer with one TA‐SeCA coated catheter segment. After a stable baseline was reached, 10 µL of GSNO (4 mM in HEPES buffer) and 100 µL of GSH (40 mM in HEPES buffer) were added to achieve a final volume of 4 mL, with final concentrations of 10 µM GSNO and 1 mM GSH. Once the current signal decayed back to the baseline, 10 µM aliquots of GSNO were repeatedly added. These included twenty times on day 1, followed by one additional cycle on day 2 and two additional cycles on day 3. Each addition was timed such that the current signal was allowed to return to the baseline before the next addition. Glass vials were covered with aluminium foil to prevent light exposure and kept at 37 °C on a hot plate with constant stirring (400 rpm) during the NO measurement. Changes in current response were recorded in a real‐time manner, and the produced NO was calculated via a calibration curve.

### Stability of Coatings

The stability of coated catheter segments to catalyze GSNO to NO was evaluated using a free radical analyzer (TBR4100, World Precision Instruments) after long‐term storage and after applying different sterilization techniques. For long‐term storage, the coated catheter segments were kept at room temperature for up to 6 months. For sterilization, two techniques were employed. First, autoclaving was performed following the Australian Standards AS/NZS 4815:2006, where the coated catheter segments were autoclaved at 121 °C and 103 kPa (1030 mb, 15 psi) for 15 min. Second, UV sterilization was carried out by placing the coated catheter segments 5 cm beneath a UV lamp (254 nm, UV lamp LF‐206.LS, 6 W, UVITEC UK) for 30 min. After these treatments, the real‐time NO generation of the coated catheter segments was determined using an electrochemical sensor as previously outlined. The stability was calculated based on the following equation:

(2) $$Stability\left(\%\right)=\frac{A_{i}}{A_{1}}\times 100$$

where A_1_ is the average NO generation by freshly prepared coated catheter segments and A_i_ is the average NO generation by coated catheter segments after sterilization or stored for different times.

### Biocompatibility and Endogenous NO Generation

HCASMCs were cultured in smooth muscle cell growth medium and subcultured when they reached a near confluent state. HCASMCs suspended in cell culture media were seeded onto 24‐well plates at a density of 20,000 cells/500 µL/well and incubated for 24 h at 37 °C in a 5% CO_2_ humidified incubator. Uncoated and coated catheter segments were all sterilized under a UV lamp for 30 min before use. Then, the sterilized catheter segments were placed into 3D‐printed inserts and incubated with cells. The control experiment involved cells that had not been incubated with catheters. After 48 and 72 h incubation, the catheters with the 3D‐printed inserts were removed and cells were washed with PBS.

For the biocompatibility test, cell media containing reagents from the LIVE/DEAD viability/cytotoxicity kit (calcein AM (2 µM) and ethidium homodimer‐1 (4 µM)) were added to each sample and incubated at 37 °C in a 5% CO_2_ humidified incubator for 45 min. After incubation, the samples were washed twice with PBS and imaged using a Zeiss LSM 800 confocal microscope. The numbers of live and dead cells were counted using Imaris software, and cell viability (%) was calculated based on the following equation:

(3) $$Cell\;Viability\;\%=\frac{Live\;Cells}{Live\;Cells+Dead\;Cells}\times 100\%$$

To assess cell proliferation, the cells were stained with the nuclear counterstain Hoechst 33342 at a 1:500 dilution. For intracellular NO detection, DAF‐FM diacetate (5 µM) was added 48 h after incubation, prior to imaging. The fluorescence intensity was processed using Imaris software.

### Hemolysis Analysis

Whole blood from C57BL/6J mice (male, 8‐10‐weeks‐old) was collected via cardiac puncture in anticoagulant tubes. The blood samples were then centrifuged at 3500 g for 5 min at room temperature to isolate red blood cells (RBCs) from plasma. After centrifugation, the RBCs were washed three times with DPBS. Then, the supernatant was discarded, and DPBS was added to obtain a 2% (v/v) RBC suspension. One uncoated or coated catheter segment was immersed in 1.5 mL of 2% RBC suspension and incubated for 2 h at room temperature, with water as a positive control and DPBS as a negative control. After incubation, all samples were centrifuged at 9000 g for 5 min, and 100 µL of the sample supernatant was collected and transferred to a 96‐well plate. The absorbance of the supernatant was measured using a microplate reader at 577 nm. The percentage hemolysis of RBCs was calculated using the following equation:

(4) $$Hemolysis\left(\%\right)=\frac{A_{1}-A_{0}}{A_{w}-A_{0}}\times 100\%$$

where A_0_ is the absorbance of blood in DPBS, A_w_ is the absorbance of blood in water, and A_1_ is the absorbance of blood treated tubes, all measured at 577 nm.

### Inflammatory Responses

RAW264.7 mouse macrophage cells (1 × 10⁵ cells/well, passage 2) were seeded onto 12‐well plates and cultured in complete medium at 37 °C in a 5% CO_2_ humidified incubator for 24 h. After incubation, the culture medium was removed, and the cells were washed three times with PBS. The cells were pre‐treated with lipopolysaccharide (LPS, 20 ng mL^−1^) for 3 h. Following this, GSNO (30 µM) and GSH (90 µM) were added in the presence or absence of uncoated or coated catheter segments. After 24 h of incubation, supernatants were collected via centrifugation at 330 *g* for 7 min. Cytokine levels (IL‐6 and TNF‐α) released from the macrophages were measured using ELISA kits, following the manufacturer's instructions (R&D Systems, USA). Additionally, cumulative NO concentrations from the supernatants were quantified using the Griess assay. Briefly, 100 µL of each supernatant was mixed with 100 µL of Griess reagent (prepared in a 1:1 mixture of 0.1% w/v N‐(1‐Naphthyl)ethylenediamine dihydrochloride and 1% w/v sulfanilamide in 5% orthophosphoric acid) in a 96‐well plate and incubated at room temperature for 15 min. The absorbance at 540 nm was measured using a microplate reader (SpectraMax M5).

### Data and Statistical Analysis

All data were presented as mean ± standard deviation, with ≥3 independent replicates. One‐way ANOVA was conducted with Tukey post hoc analysis. Statistical difference is denoted when *p* < 0.05. *p < 0.05, **p < 0.01, ***p < 0.001, and ****p < 0.0001.

## Conflict of Interest

The authors declare no conflict of interest.

## Supporting information



Supporting Information

## Data Availability

The data that support the findings of this study are available from the corresponding author upon reasonable request.
