# Supplementary material for: Nitric Oxide‐Releasing Catheters with Phenol‐Amine Catalytic Coatings for Improved Anti‐Inflammatory Performance
Source: Adv Healthc Mater. 2025 Aug 29;14(31):e00457. doi: 10.1002/adhm.202500457 (PMC12683202; doi:10.1002/adhm.202500457)
Supplement: Supplementary file 1 — Supporting Information [file ADHM-14-0-s001.docx]

**Supporting Information**

**Nitric Oxide-Releasing Catheters with Phenol-Amine Catalytic Coatings for Improved Anti-Inflammatory Performance**

Qingqing Fan^1,2^, Shu Geng^1^, Tanveer A. Tabish^3^, Kang Lin^1^, Yiyi Yin^1^, Siti Nur Asyura Adzlan^1^, Federico Mazur^1^, and Rona Chandrawati^1*^

^1^School of Chemical Engineering and Australian Centre for Nanomedicine (ACN), The University of New South Wales, Sydney, NSW 2052, Australia

^2^Department of Chemical Engineering, The University of Melbourne, Parkville, VIC 3010, Australia

^3^Division of Cardiovascular Medicine, Radcliffe Department of Medicine, British Heart Foundation (BHF) Centre of Research Excellence, University of Oxford, Headington, Oxford OX3 7BN, United Kingdom

*Corresponding author: [rona.chandrawati@unsw.edu.au](mailto:rona.chandrawati@unsw.edu.au)

**Table S1.** Contact angle (CA) of uncoated and coated catheter segments at various TA and SeCA coating ratios.

|  | CA left [°] | CA right [°] | CA mean [°] |
| --- | --- | --- | --- |
| Uncoated catheter segments | 105.09 ± 1.67 | 105.53 ± 2.15 | 105.31 ± 1.90 |
| 1:0.1 | 101.42 ± 0.80 | 102.02 ± 0.77 | 101.72 ± 0.77 |
| 1:0.5 | 100.39 ± 0.74 | 100.26 ± 0.88 | 100.33 ± 0.77 |
| 1:1 | 105.14 ± 0.77 | 105.25 ± 1.11 | 105.19 ± 0.90 |
| 1:2 | 102.86 ± 1.40 | 102.89 ± 1.20 | 102.88 ± 1.21 |
| 1:4 | 101.65 ± 2.24 | 102.37 ± 2.47 | 102.01 ± 2.35 |
| 1:8 | 106.74 ± 9.34 | 106.53 ± 9.13 | 106.63 ± 9.22 |


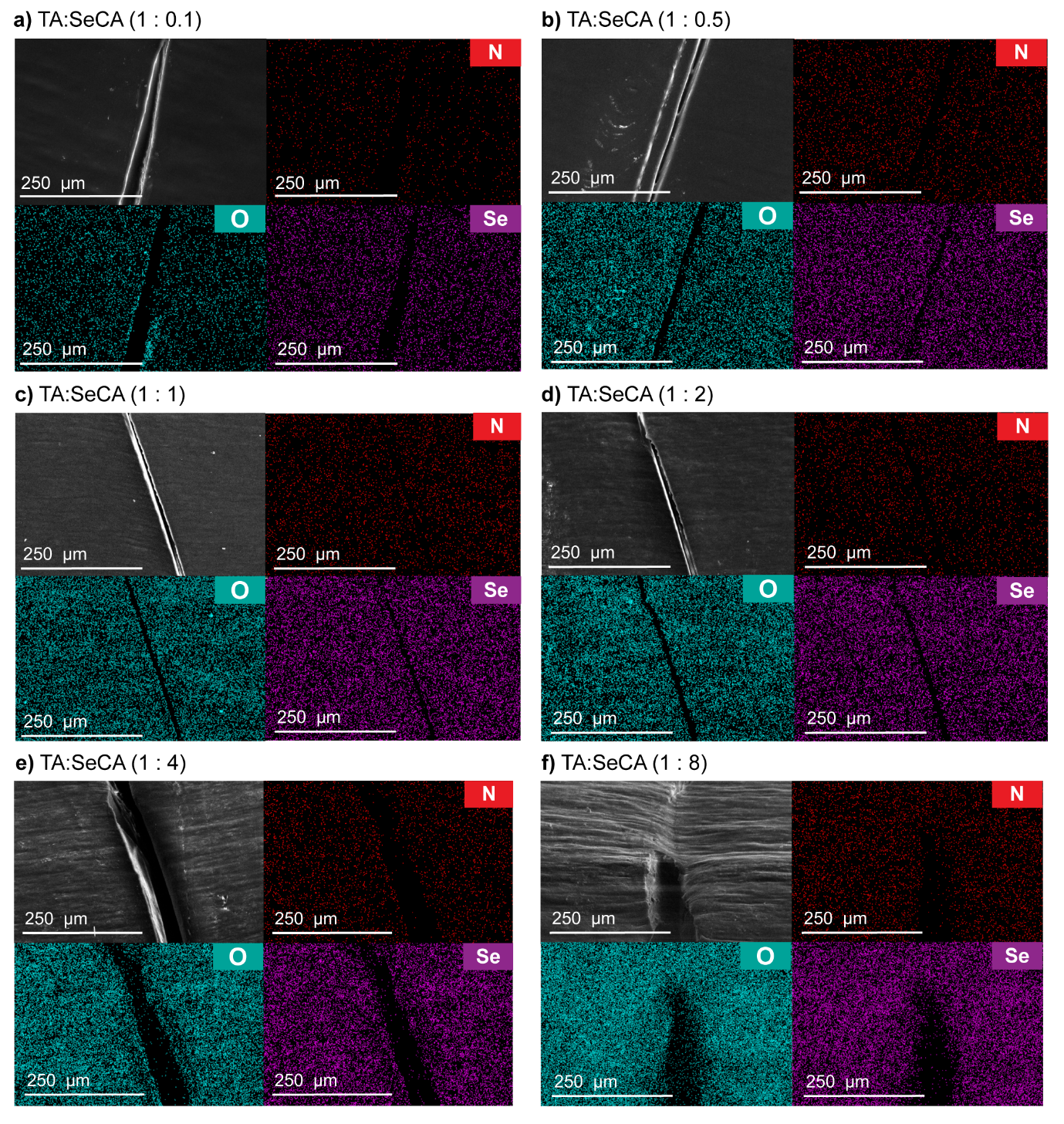


**Figure S1.** Elemental distribution mapping using EDS for coated catheter segments at various TA and SeCA coating ratios: (a) 1:0.1, (b) 1:0.5, (c) 1:1, (d) 1:2, (e) 1:4, and (f) 1:8. The dark line visible in each figure represents a scratch test performed to observe a clear boundary, providing a reference point for EDS mapping measurements and ensuring accurate elemental analysis across the coated surface.


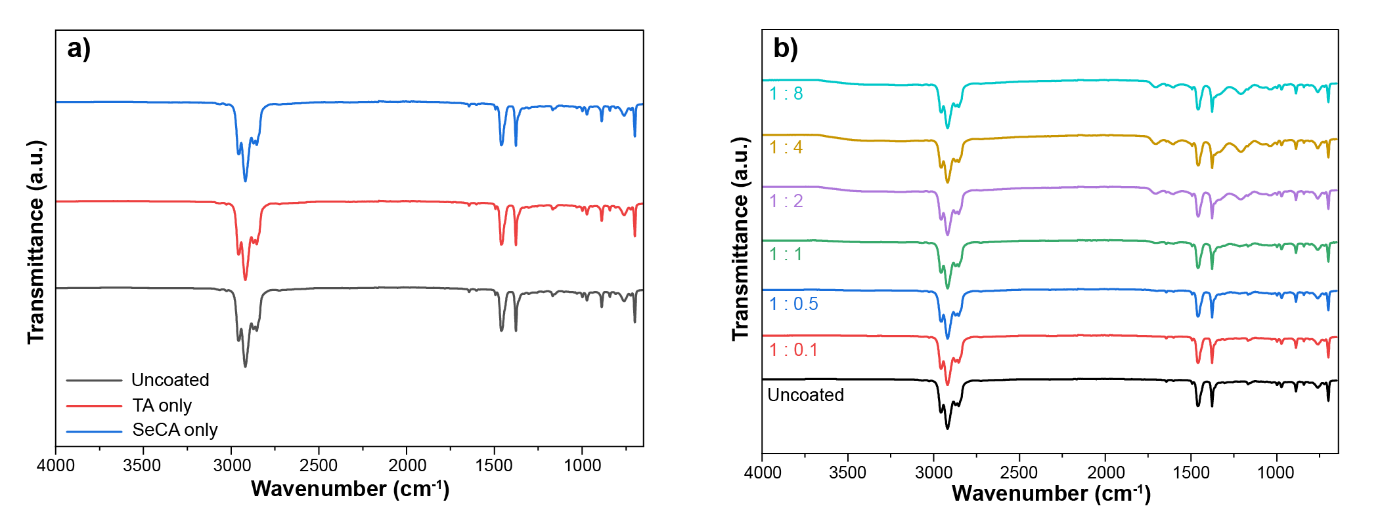


**Figure S2.** (a) FT-IR spectra of uncoated catheter segments as well as catheter segments coated with SeCA only or TA only. (b) FT-IR spectra of uncoated and coated catheter segments at various TA:SeCA coating ratios (1:0.1, 1:0.5, 1:1, 1:2, 1:4, and 1:8).


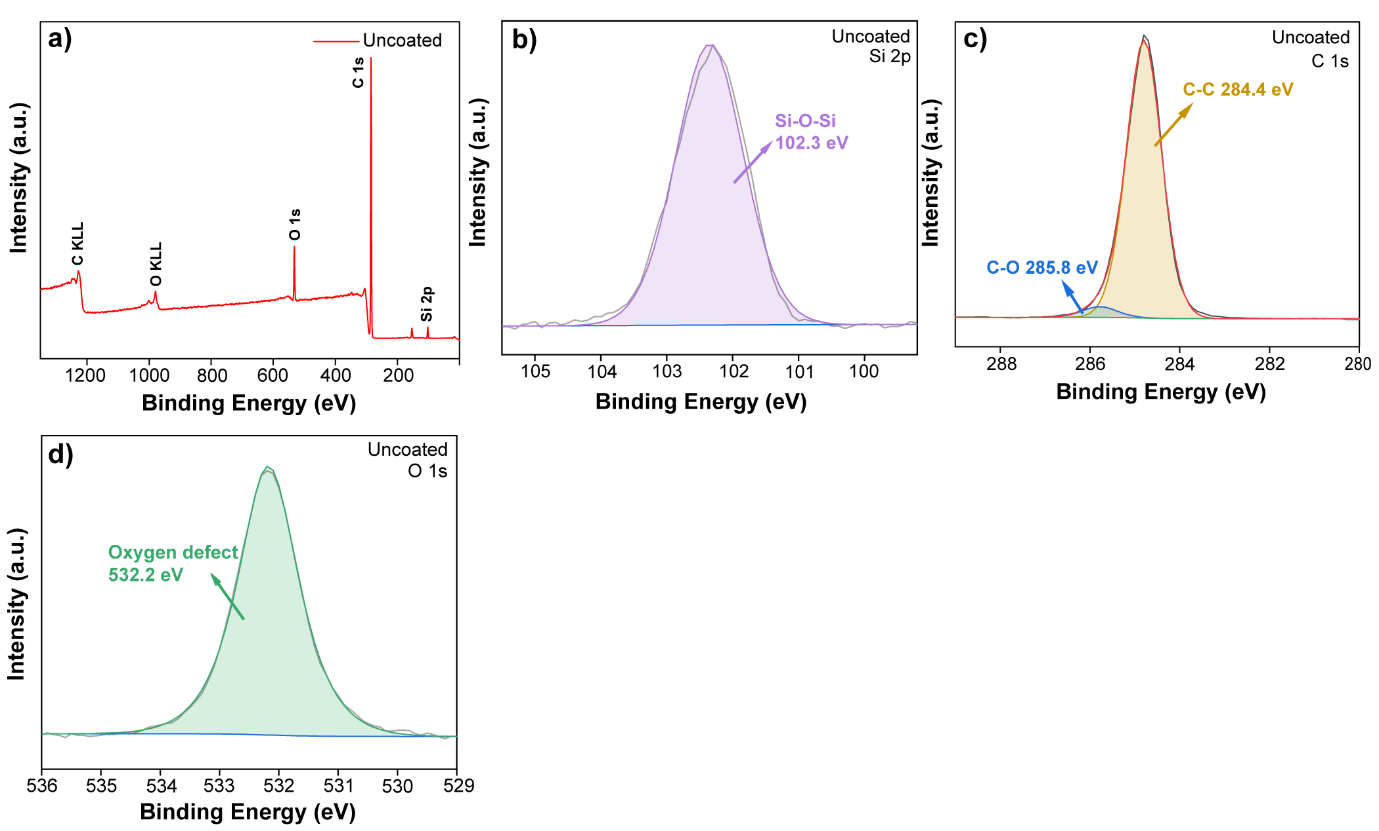


**Figure S3.** XPS spectra for uncoated catheter segments: (a) survey, (b) Si 2p*, (c) C 1s, (d) O 1s. *Si 2p signals originate from instrumental background contamination (XPS sample holder/stage), not from coating or catheter materials.


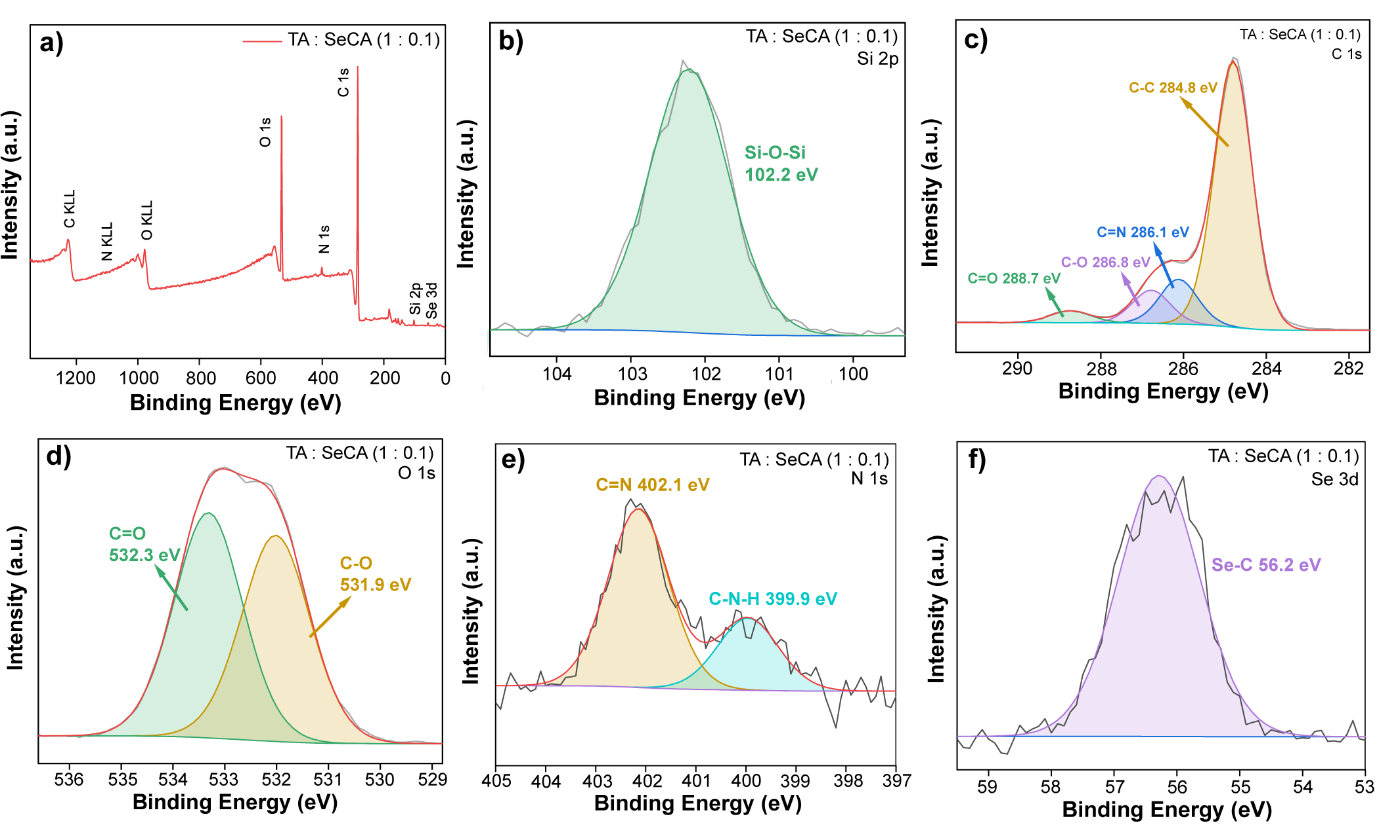


**Figure S4.** XPS spectra for coated catheter segments coating ratio at 1:0.1: (a) survey, (b) Si 2p*, (c) C 1s, (d) O 1s, (e) N 1s, and (f) Se 3d. *Si 2p signals originate from instrumental background contamination (XPS sample holder/stage), not from coating or catheter materials.


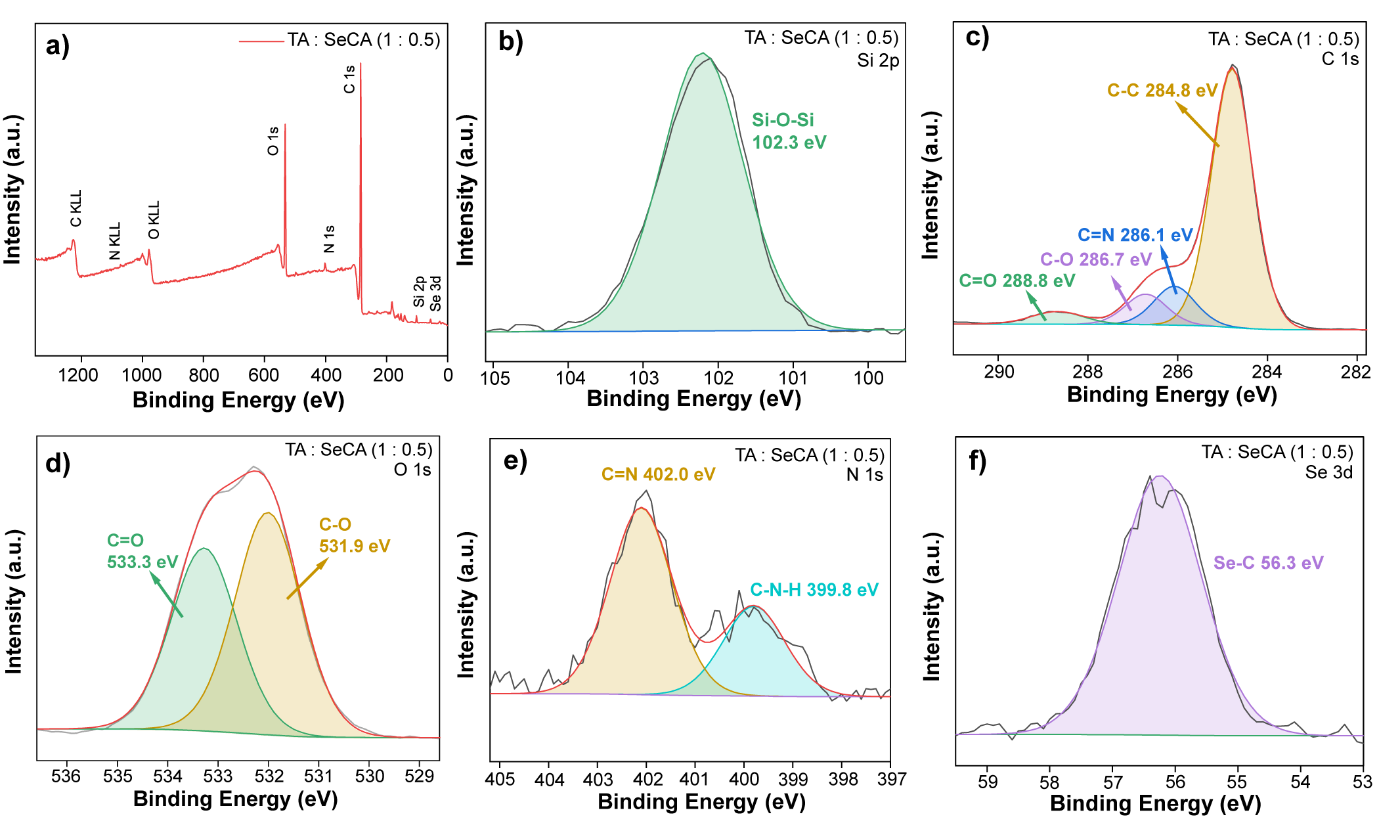


**Figure S5.** XPS spectra for coated catheter segments coating ratio at 1:0.5: (a) survey, (b) Si 2p*, (c) C 1s, (d) O 1s, (e) N 1s, and (f) Se 3d. *Si 2p signals originate from instrumental background contamination (XPS sample holder/stage), not from coating or catheter materials.


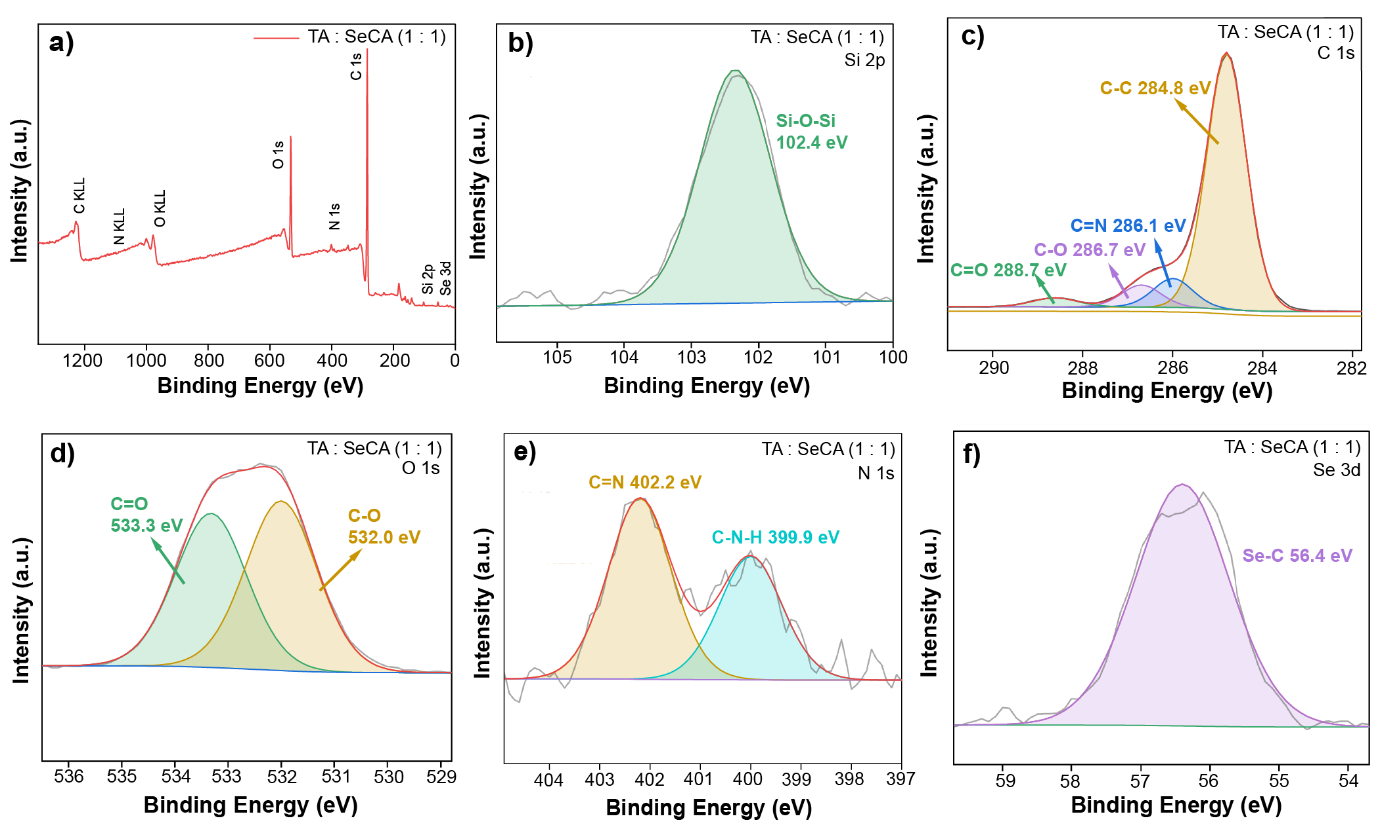


**Figure S6.** XPS spectra for coated catheter segments coating ratio at 1:1: (a) survey, (b) Si 2p*, (c) C 1s, (d) O 1s, (e) N 1s, and (f) Se 3d. *Si 2p signals originate from instrumental background contamination (XPS sample holder/stage), not from coating or catheter materials.


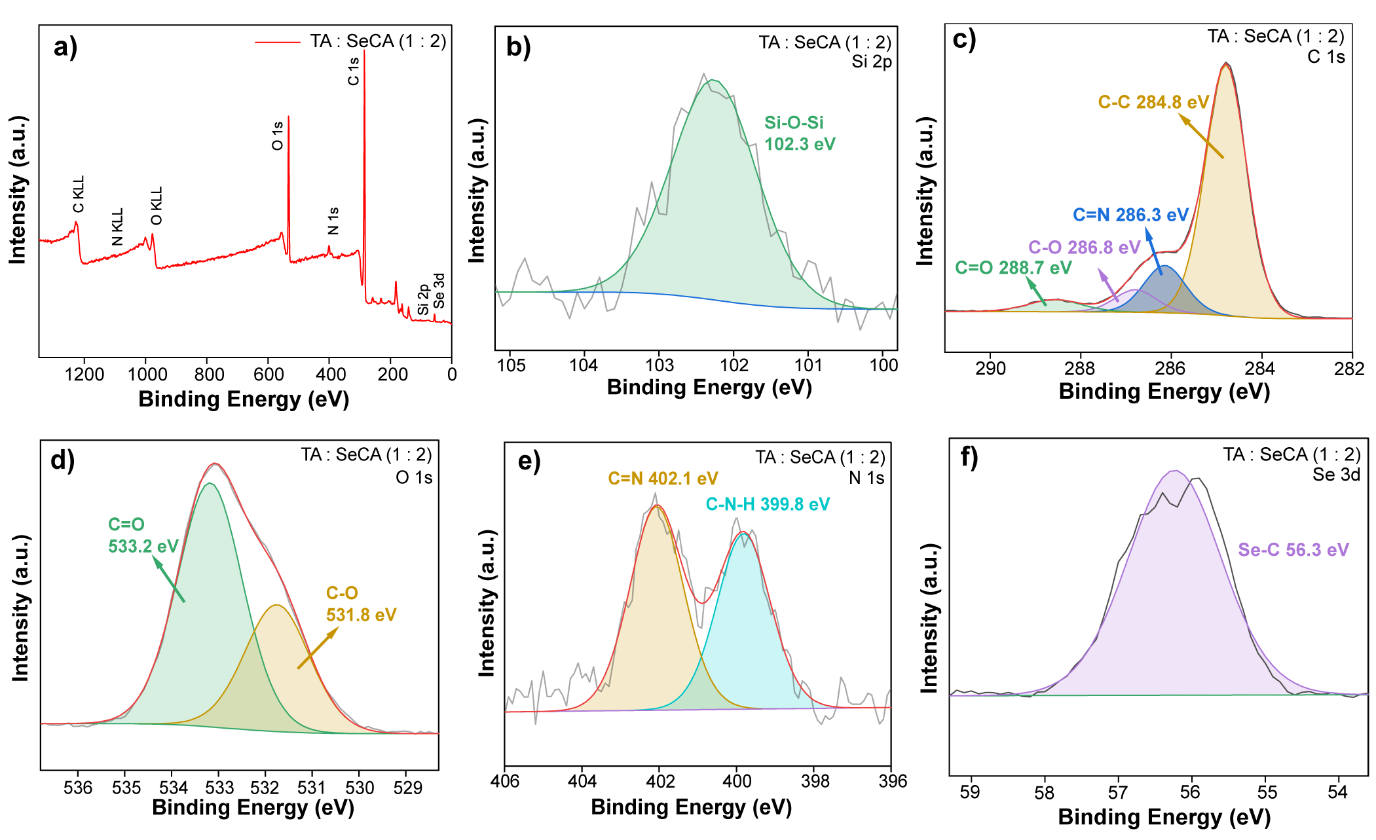


**Figure S7.** XPS spectra for coated catheter segments coating ratio at 1:2: (a) survey, (b) Si 2p*, (c) C 1s, (d) O 1s, (e) N 1s, and (f) Se 3d. *Si 2p signals originate from instrumental background contamination (XPS sample holder/stage), not from coating or catheter materials.


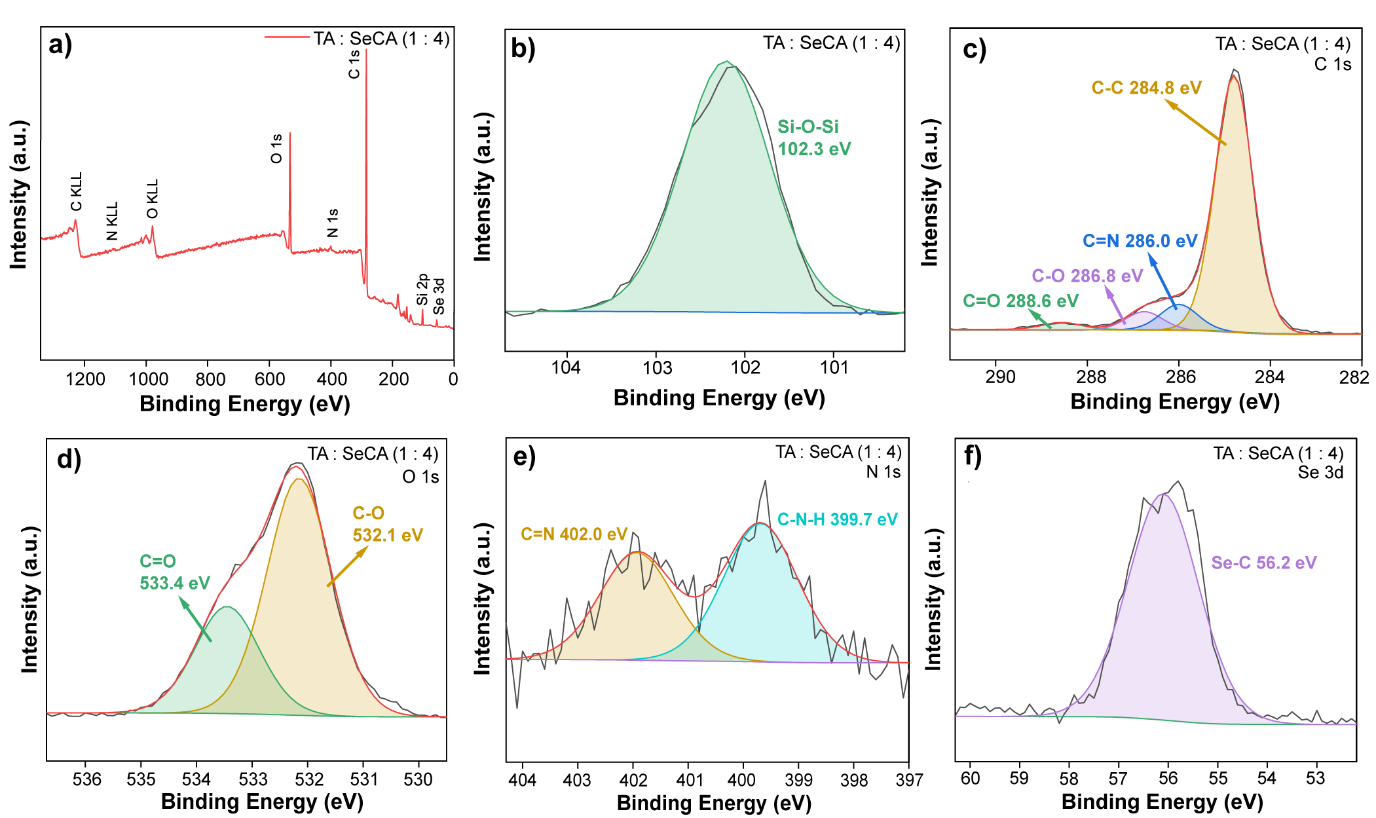


**Figure S8.** XPS spectra for coated catheter segments coating ratio at 1:4: (a) survey, (b) Si 2p*, (c) C 1s, (d) O 1s, (e) N 1s, and (f) Se 3d. *Si 2p signals originate from instrumental background contamination (XPS sample holder/stage), not from coating or catheter materials.


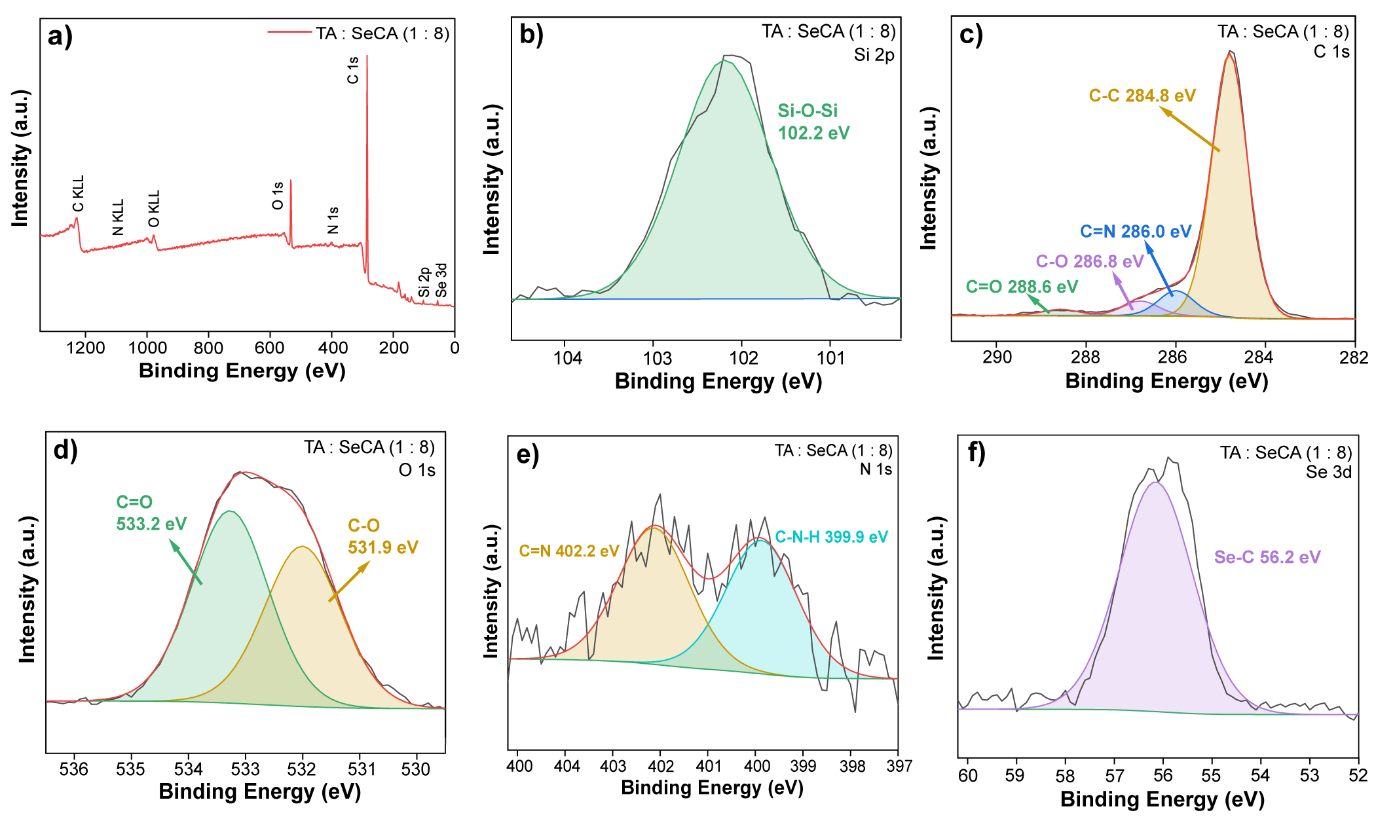


**Figure S9.** XPS spectra for coated catheter segments coating ratio at 1:8: (a) survey, (b) Si 2p*, (c) C 1s, (d) O 1s, (e) N 1s, and (f) Se 3d. *Si 2p signals originate from instrumental background contamination (XPS sample holder/stage), not from coating or catheter materials.

**Table S2.** XPS analysis of Se atomic % for catheter segments at different coating ratios of TA and SeCA.

| **Coating Ratio** | **Se atomic %** |
| --- | --- |
| **Uncoated** | Undetected |
| **1:0.1** | 0.48 |
| **1:0.5** | 0.66 |
| **1:1** | 0.70 |
| **1:2** | 1.09 |
| **1:4** | 1.16 |
| **1:8** | 1.02 |


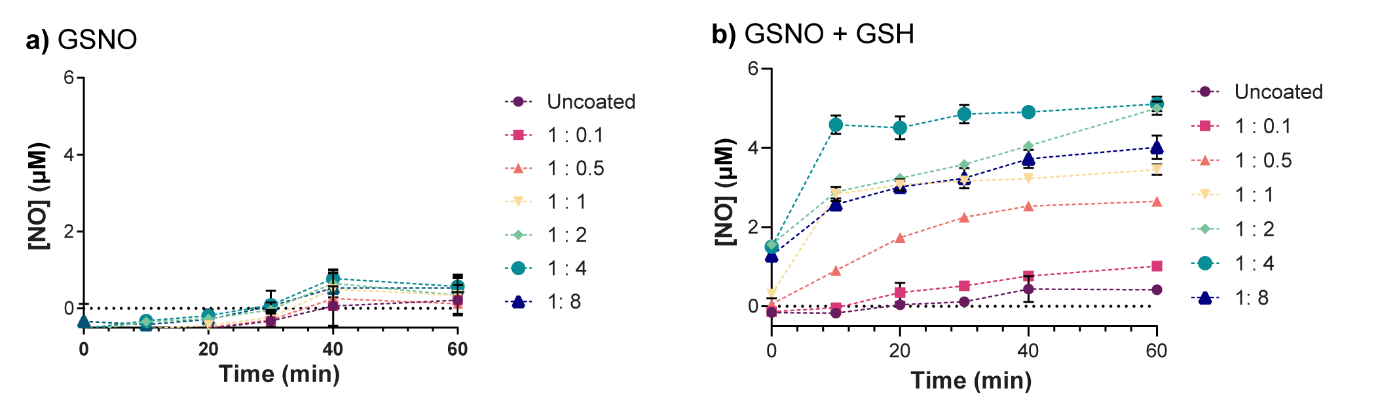


**Figure S10.** Time dependent NO generation from TA-SeCA coated catheter segments (at various molar ratios 1:0.1 to 1:8) when exposed to GSNO (10 μM) in the (a) absence or (b) presence of GSH (1 mM). All reactions were performed in HEPES buffer (pH 7.4) at 37 °C. Values represent mean ± standard deviation, n = 3.


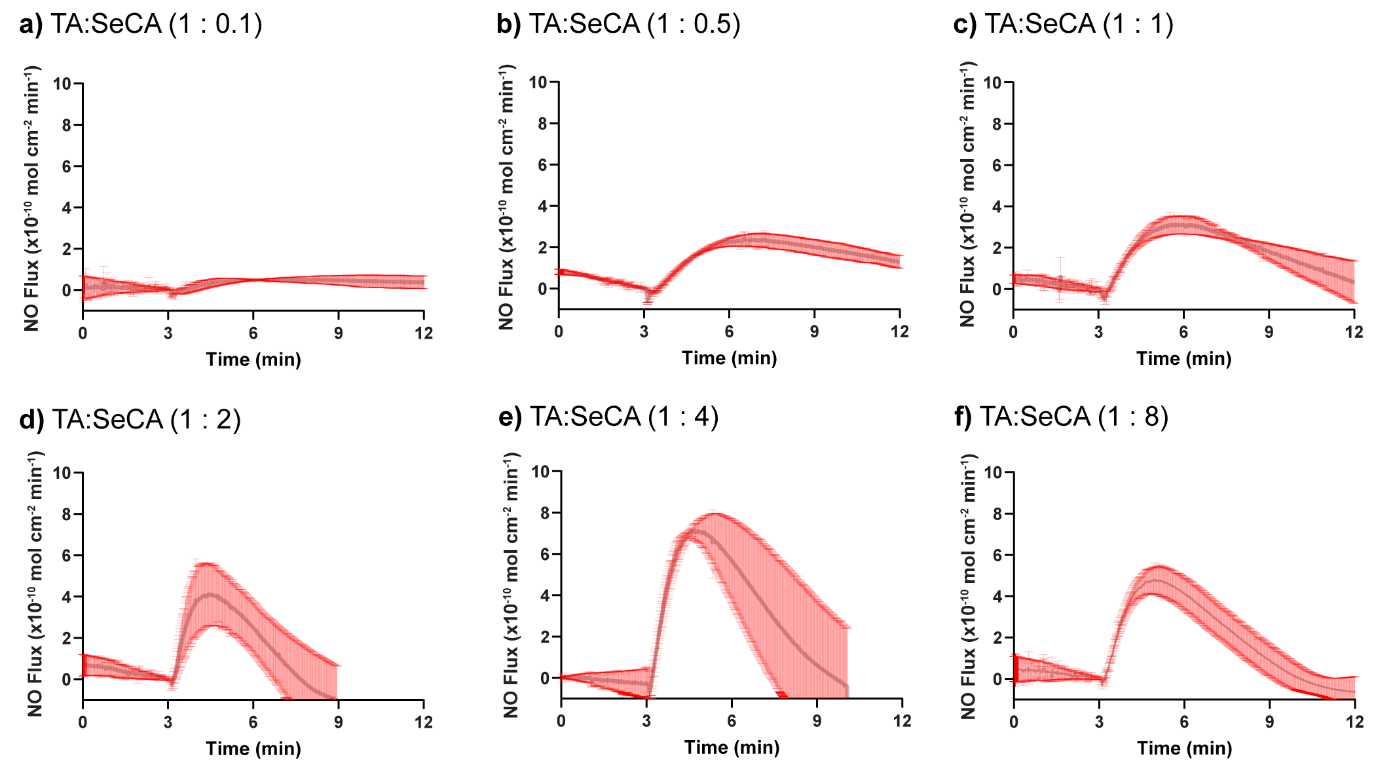


**Figure S11**. Real-time NO flux from TA:SeCA coated catheter segments at various molar ratios: (a) 1:0.1, (b) 1:0.5, (c) 1:1, (d) 1:2, (e) 1:4, and (f) 1:8, when exposed to GSNO (10 μM) in the presence of GSH (1 mM). All reactions were performed in HEPES buffer (pH 7.4) at 37 °C. Values represent mean ± standard deviation, n = 3.


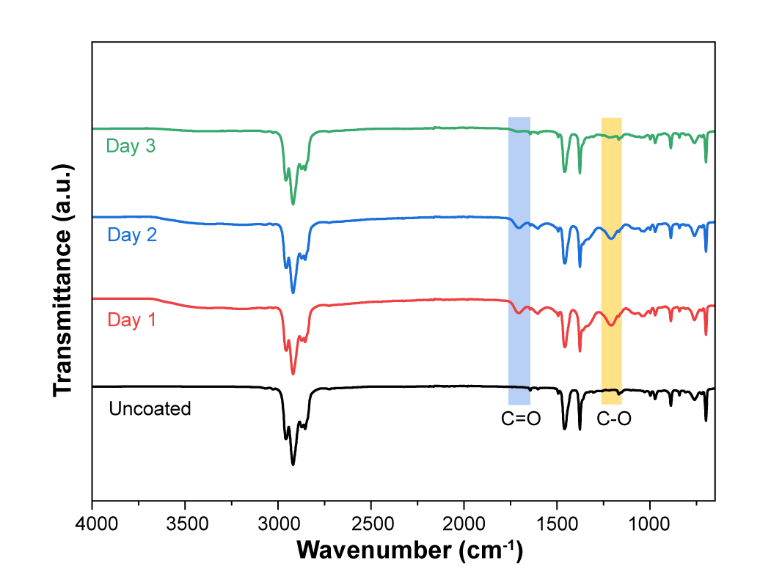


**Figure S12**. FT-IR spectra of the coated catheter segments recorded over a 72-hour period.


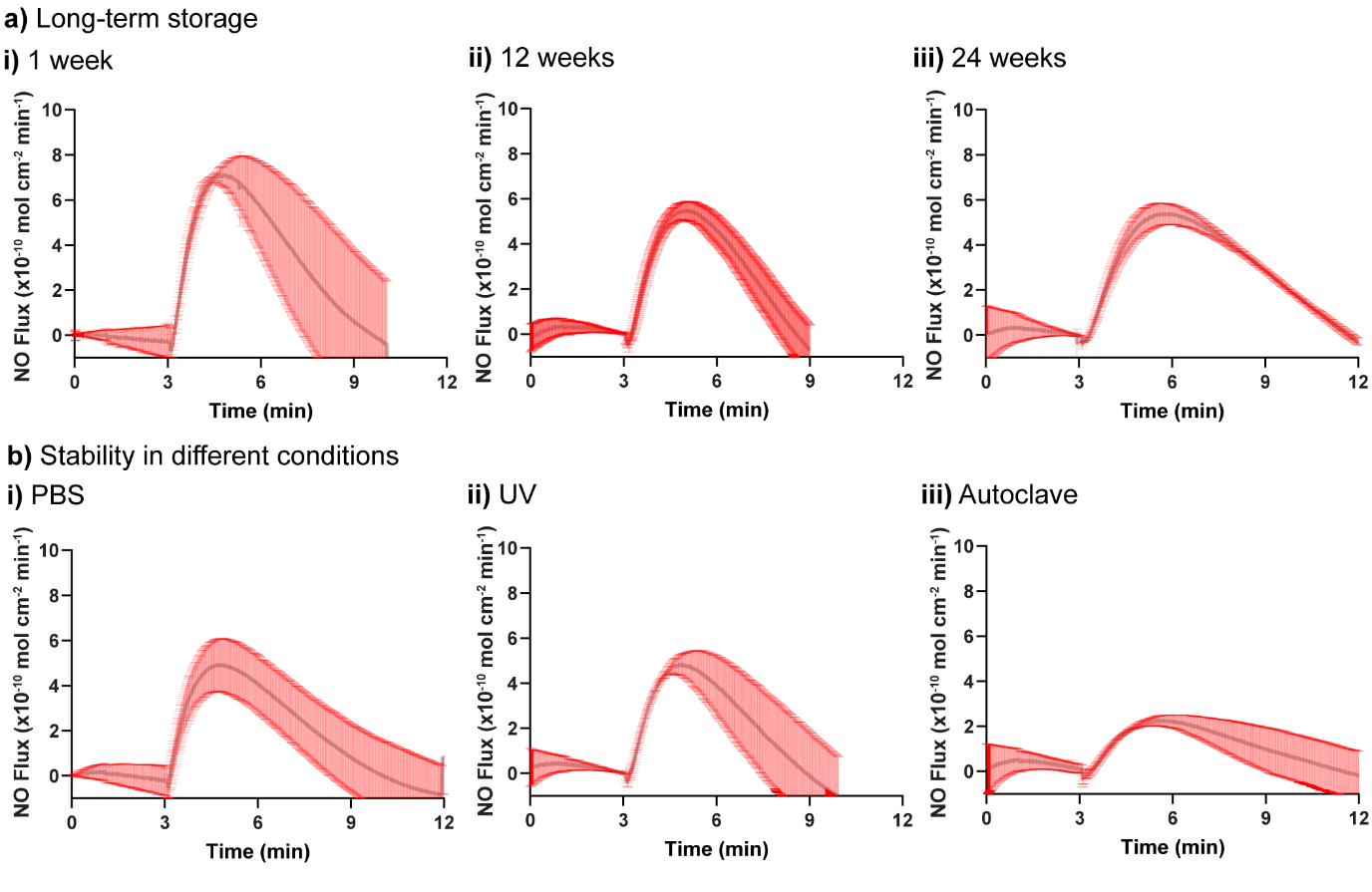


**Figure S13**. Real-time NO flux of a coated catheter segment at a TA:SeCA molar ratio of 1:4, evaluated under different conditions: (a) after long-term storage for (i) 1 week, (ii) 12 weeks, and (iii) 24 weeks in HEPES (10 mM, pH 7.4); and (b) stability tests including NO release in (i) PBS (10 mM, pH 7.4), and NO release after (ii) UV-irradiation and (iii) autoclave sterilization in HEPES (10 mM, pH 7.4). All experiments were exposed to GSNO (10 μM) in the presence of GSH (1 mM) at 37 °C. Values represent mean ± standard deviation, n = 3.


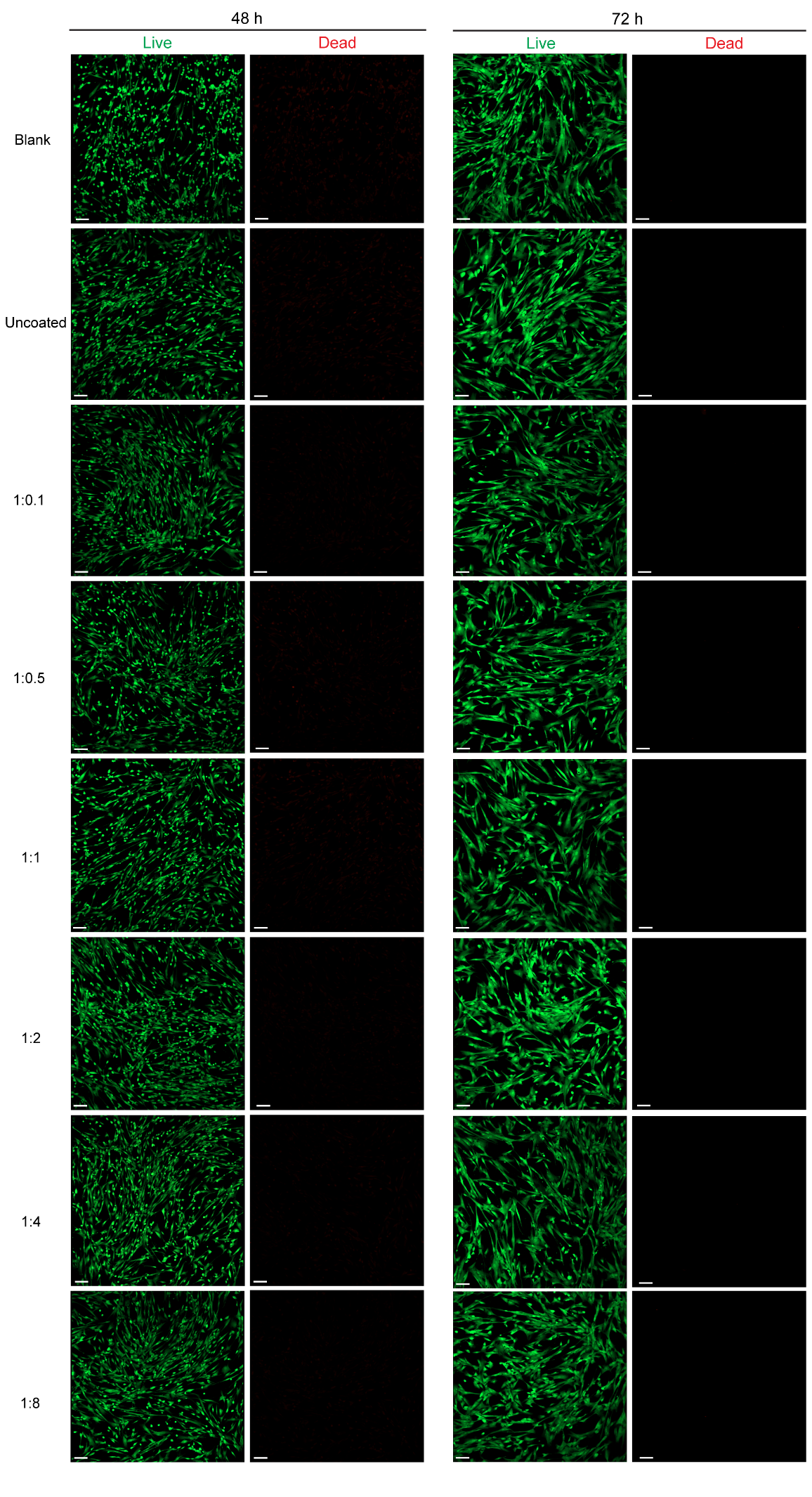


**Figure S14**. Fluorescence microscopy images of Live/Dead assay. HCASMCs were cultured for 48 and 72 h with uncoated and TA:SeCA coated catheter segments at various molar ratios (1:0.1, 1:0.5, 1:1, 1:2, 1:4, 1:8). Green indicates live cells and red indicates dead cells. Scale bars: 100 μm.


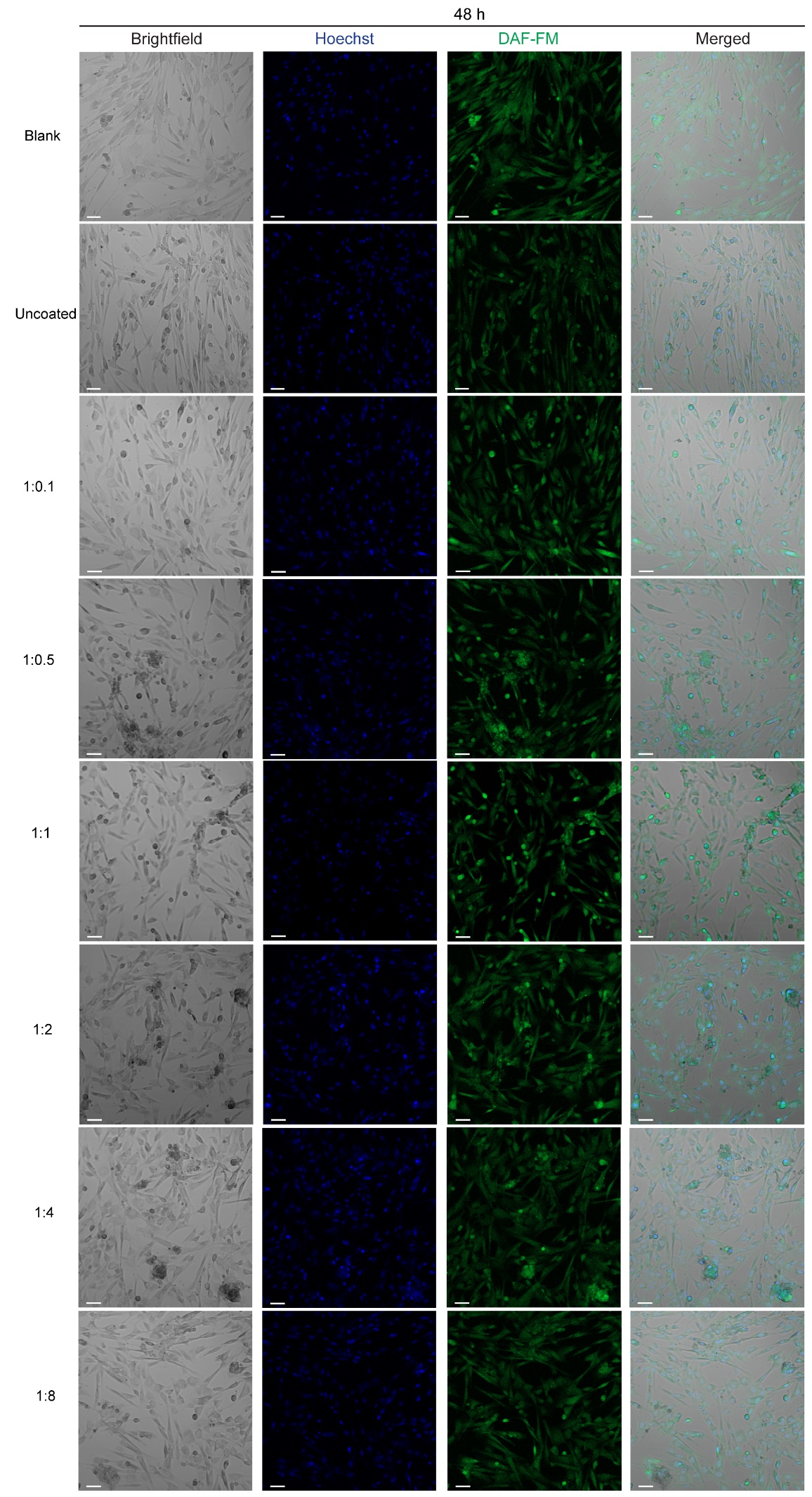


**Figure S15**. Fluorescence and brightfield microscopy images of HCASMCs stained with Hoechst and DAF-FM diacetate. HCASMCs were cultured for 48 h with uncoated and TA:SeCA coated catheter segments at various molar ratios (1:0.1, 1:0.5, 1:1, 1:2, 1:4, 1:8). Blue fluorescence indicates cell nuclei, while green fluorescence indicates NO production in live cells. Scale bars: 100 μm.


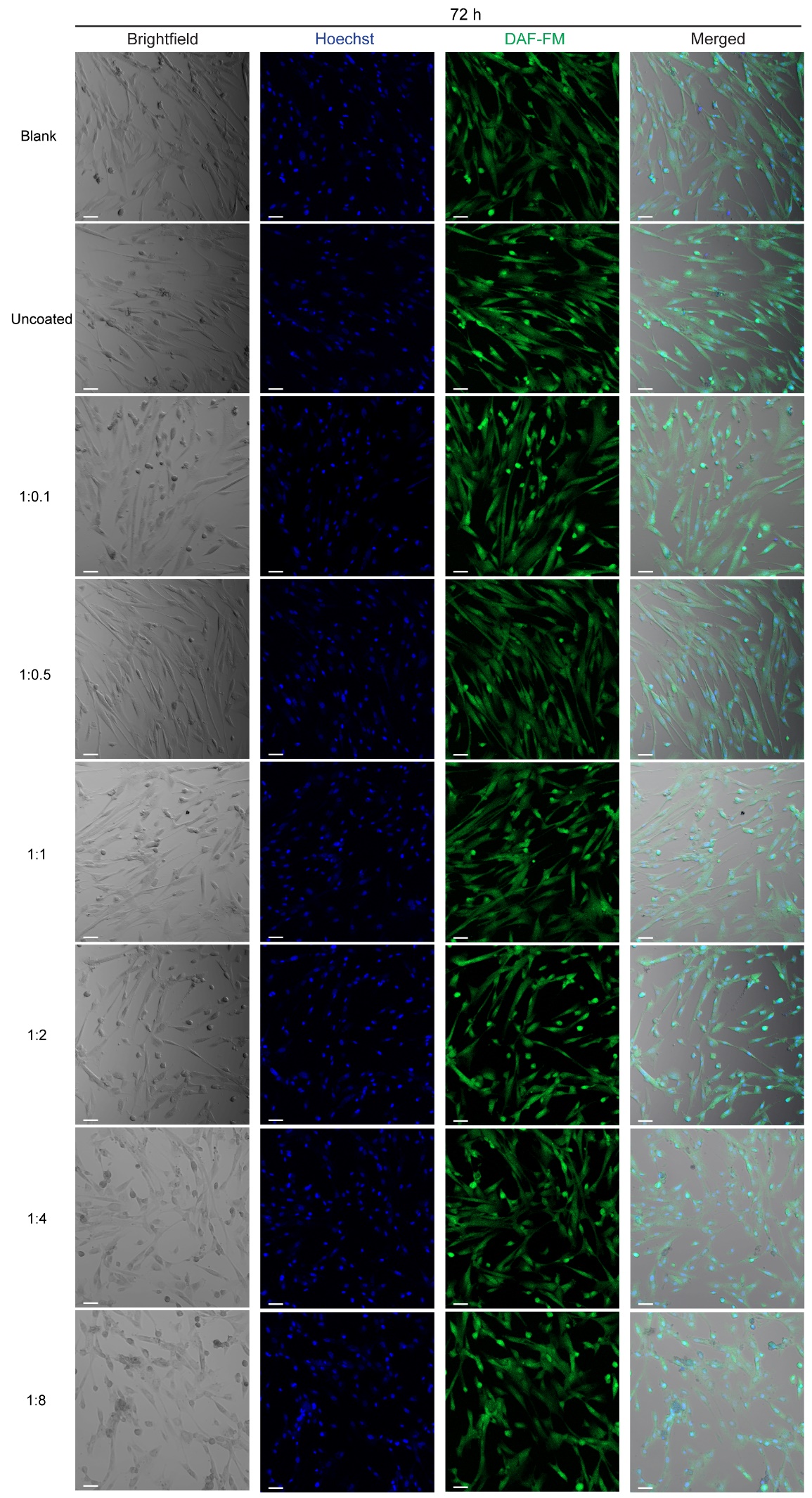


**Figure S16**. Fluorescence and brightfield microscopy images of HCASMCs stained with Hoechst and DAF-FM diacetate. HCASMCs were cultured for 72 h with uncoated and TA:SeCA coated catheter segments at various molar ratios (1:0.1, 1:0.5, 1:1, 1:2, 1:4, 1:8). Blue fluorescence indicates cell nuclei, while green fluorescence indicates NO production in live cells. Scale bars: 100 μm.
